# Evidence Mapping of 23 Systematic Reviews of Traditional Chinese Medicine Combined With Western Medicine Approaches for COVID-19

**DOI:** 10.3389/fphar.2021.807491

**Published:** 2022-02-07

**Authors:** Ting Zhang, Xuechao Li, Yamin Chen, Liang Zhao, Jinhui Tian, Junhua Zhang

**Affiliations:** ^1^ Evidence-Based Medicine Center, Tianjin University of Traditional Chinese Medicine, Tianjin, China; ^2^ Clinical Research Center, Affiliated Hospital of Shannxi University of Chinese Medicine, Xianyang, China; ^3^ Evidence-Based Nursing Center, School of Nursing, Lanzhou University, Lanzhou, China; ^4^ Institute of Evidence-Based Medicine Center, School of Basic Medical Sciences, Lanzhou University, Lanzhou, China

**Keywords:** COVID-19, traditional Chinese medicine combined with Western medicine approaches, systematic reviews, evidence mapping, AMSTAR 2, ROBIS, grade

## Abstract

**Background:** Coronavirus disease 2019 (COVID-19) has already spread around the world. The modality of traditional Chinese medicine (TCM) combined with Western medicine (WM) approaches is being used to treat COVID-19 patients in China. Several systematic reviews (SRs) are available highlighting the efficacy and safety of TCM combined with WM approaches in COVID-19 patients. However, their evidence quality is not completely validated.

**Purpose:** We aimed to assess the methodological quality and the risk of bias of the included SRs, assess the evidence quality of outcomes, and present their trends and gaps using the evidence mapping method.

**Methods:** PubMed, Cochrane Library, Embase, CNKI, CBM, and Wanfang Data were searched from inception until March 2021 to identify SRs pertaining to the field of TCM combined with WM approaches for COVID-19. The methodological quality of the SRs was assessed using the Assessment of Multiple Systematic Reviews 2 (AMSTAR 2), the risk of bias of the included SRs was assessed with the Risk of Bias in Systematic Review (ROBIS) tool, and the evidence quality of outcomes was assessed using the Grading of Recommendations Assessment, Development and Evaluation (GRADE) system.

**Results:** In total, 23 SRs were found eligible. Twenty-one were rated of moderate confidence by AMSTAR 2, while 12 were rated at low risk using the ROBIS tool. In addition, most outcomes were graded as having moderate quality using the GRADE system. We found that the combined use of TCM and WM approaches could improve the CT recovery rate, effective rate, viral nucleic acid negative conversion rate, and the disappearance rate of fever, cough, and shortness of breath. Also, these approaches could decrease the conversion rate from mild to critical, white blood cell counts, and lymphocyte counts and shorten the time to viral assay conversion and the length of hospital stay.

**Conclusion:** TCM combined with WM approaches had advantages in efficacy, laboratory, and clinical symptom outcomes of COVID-19, but the methodological deficiencies of SRs should be taken into consideration. Therefore, to better guide clinical practice in the future, the methodological quality of SRs should still be improved, and high-quality randomized controlled trials (RCTs) and observational studies should also be carried out.

## Introduction

Coronavirus disease 2019 (COVID-19) is a viral disease caused by severe acute respiratory syndrome coronavirus-2 (SARS-CoV-2). The disease was named COVID-19 by the World Health Organization (WHO) on February 11, 2020 (WHO, 2020a; WHO, 2020b; WHO, 2020c; Nie et al., 2020). The main symptoms of COVID-19 patients are fever, cough, shortness of breath, and acute respiratory distress syndrome, which eventually lead to death in several cases (Huang et al., 2020). Due to its highly contagious nature, COVID-19 has spread rapidly throughout the world within a few months after the identification of its first case in December 2019. COVID-19 was declared a public health emergency of international concern and a pandemic on March 11, 2020 (WHO, 2020). This disease had already affected the economy of various countries and deeply impacted the daily life of people (BBC News, 2020). In China, COVID-19 is well under control, possibly due to the use of traditional Chinese medicine (TCM) combined with Western medicine (WM) approaches and strict public quarantine measures. In Jiangxia Fangcang TCM Hospital, among 564 confirmed cases, 482 were cured and 82 complicated cases were transferred to other designated hospitals. No patients turned from mild to critical, and no nurses and doctors encountered COVID-19 in the above-mentioned hospital (Jin Y. H. et al., 2020).

Moreover, increasing evidence based on systematic reviews (SRs) of TCM combined with WM approaches (TCM + WM) for COVID-19 have found that TCM + WM approaches could improve the clinical efficacy, cure rate, lung CT readings, and length of hospital stay. These approaches can further alleviate fever reduction time, rate of cough, and fatigue and improve laboratory indicators such as white blood cell counts, lymphocyte counts, and C-reactive protein levels (Lin et al., 2020; Xiong et al., 2020; Liu M. et al., 2021).

SRs serve as the basis for the development of practice guidelines to improve the quality of decision-making for patient care, and their reliability is largely determined by their quality (Wallace et al., 2012; Li et al., 2021). Evidence mapping is a comprehensive evidence-based research method that is being increasingly used to systematically and rapidly identify, evaluate, organize, and present existing evidence to give directions for future research while addressing gaps and promoting scientific research and decision-making (Anaya et al., 2019; Lam et al., 2019; Sun Y. et al., 2020). Because of these advantages, we used the evidence mapping method to present the trends and gaps in the risk of bias of the included SRs and the evidence quality of outcomes of TCM + WM approaches for COVID-19. Our findings are expected to promote evidence-based decision-making.

## Methods

The present study was performed according to the guidelines of Preferred Reporting Items for Overviews of Systematic Reviews including a harms checklist (PRIO-harms) (Bougioukas et al., 2018).

### Inclusion and Exclusion Criteria

SRs of randomized controlled trials (RCTs) or observational studies (OSs) or both for COVID-19 were included. At the same time, the quantitative method of meta-analysis was used to analyze the data of the included SRs. Patients diagnosed with COVID-19 were enrolled in this study, with no restrictions of age, gender, and nationality. TCM (Chinese herbs, herbal decoctions, and Chinese patent medicine) combined with WM approaches was used as the intervention. WM approaches (including conventional medications, such as antibacterial, antiviral, hormone therapy, and respiratory support) were used for comparison. In our analysis, we investigated the efficacy, laboratory, safety, and clinical symptom outcomes. We excluded SRs that were in languages other than English or Chinese and also those with the full text unavailable.

### Search Strategy

We searched the following six databases: PubMed, Cochrane Library, Embase, China National Knowledge Infrastructure (CNKI), China Biology Medicine (CBM), and Wanfang Data. The search period for the included studies was from the inception of each database until March 25, 2021. The search terms included “Traditional Chinese Medicine,” “Chinese drug,” “2019-nCoV,” “COVID-19,” ”SARS-CoV-2,” “systematic review,” and “meta-analysis.” The search strategy for the PubMed database is presented in *Supplementary Material S1*. Additionally, we also searched Google Scholar and manually examined the reference lists to identify additional eligible studies.

### Study Selection and Data Extraction

Endnote X9 (Clavirate Analytics, Spring Garden, PA, USA) software was used to manage the identified records and remove duplicates. Two reviewers (TZ and XCL) independently screened the titles and abstracts to determine potential studies as per the inclusion and exclusion criteria. Then, the full texts of the potential studies were obtained and further screened to identify eligible SRs. Details about the authors, publication year, type of disease, diagnostic criteria, study design, sample size, interventions and comparisons, quality assessment methods, and outcomes were extracted by two reviewers independently using a pre-designed extraction form. Any disagreements were resolved by consensus or by consulting a third reviewer (JZ).

### Methodological Quality Assessment of the Included SRs

Two reviewers (TZ and XL) separately assessed the methodological quality of the included SRs using the Assessment of Multiple Systematic Reviews 2 (AMSTAR 2) (Shea et al., 2017). Any disagreements were resolved by discussion or by consulting a third reviewer (JZ). Each of the 16 items was classified as “Yes,” “No,” or “Partial Yes.” The overall confidence in the results of SRs was categorized into four levels: high, moderate, low, or very low (Shea et al., 2017). Detailed information on the AMSTAR 2 checklist is shown in *Supplementary Material S2*.

### Assessment of Risk of Bias of the Included SRs

Two reviewers (TZ and XL) independently assessed the risk of bias of the included SRs using the Risk of Bias in Systematic Review (ROBIS) tool (Whiting et al., 2016). Any disagreements were resolved by mutual discussion or by consulting a third reviewer (JZ). The ROBIS tool consisted of four domains: 1) study eligibility criteria; 2) identification and selection of studies; 3) data collection; and 4) study appraisal and synthesis and findings. Each domain had five to six questions that were answered as “Yes,” “Probably Yes,” “Probably No,” “No,” and “No Information.” If all answers were “Yes” or “Probably Yes,” then that domain was considered as “Low Risk.” If the answers were “No” or “Probably No,” then that domain was considered as “High Risk.” The remaining domains were considered as “Unclear Risk” (Whiting et al., 2016).

### Summary of the Risk of Bias of the RCTs and OSs Included in SRs

We summarized the risk of bias of the included RCTs and OSs. Their results are presented as frequencies and percentages.

### Assessment of the Evidence Quality of Outcomes

Two reviewers (TZ and XL) independently assessed the evidence quality of outcomes using the Grading of Recommendations Assessment, Development and Evaluation (GRADE) system (Guyatt et al., 2008; Higgins et al., 2021). Any disagreements were resolved by mutual consensus or by consulting a third reviewer (JZ). In the assessment, if one outcome was included in the RCTs or OSs, the GRADE approach was used to downgrade or upgrade the evidence. Five downgraded factors were taken into consideration in the evidence assessment, including the risk of bias, inconsistency, indirectness, imprecision, and publication bias. The three upgrading factors were larger effect, dose–response gradient, and plausible confounding. Furthermore, if one outcome was included in both RCTs and OSs, the corresponding RCTs were only used to evaluate the quality of evidence. The overall quality of evidence was categorized as high, moderate, low, or very low.

### Data Synthesis and Analysis

Microsoft Excel 2019 was used to extract, manage, and analyze the data and to generate figures.

The frequency and percentage of descriptive statistics were used to analyze the data in this study. A bar chart was utilized to show the methodological quality results of the included SRs and the summary risk of bias of the RCTs and OSs included in the SRs. In addition, a bubble plot was used to display information in four dimensions in order to present the result of risk of bias of the included SRs and the evidence quality of outcomes (Li et al., 2021; Sun et al., 2020). Details about presenting risk of bias of the included SRs are as follows: a) each bubble represents the result of risk bias; b) green color represents low risk, yellow color represents unclear risk, and red color represents a high risk; c) ROBIS tool items are represented on the lateral axis; and d) the included SRs are represented on the vertical axis. Details about presenting the evidence quality of outcomes are as follows: a) each bubble size represents the number of patients included in the SR; b) different colors represent the *p*-value, green color represents *p* < 0.05, and red color represents *p* > 0.05; c) outcomes are represented on the lateral axis; and d) evidence quality of outcome is represented on the vertical axis.

## Results

### Literature Selection

**FIGURE 1:**
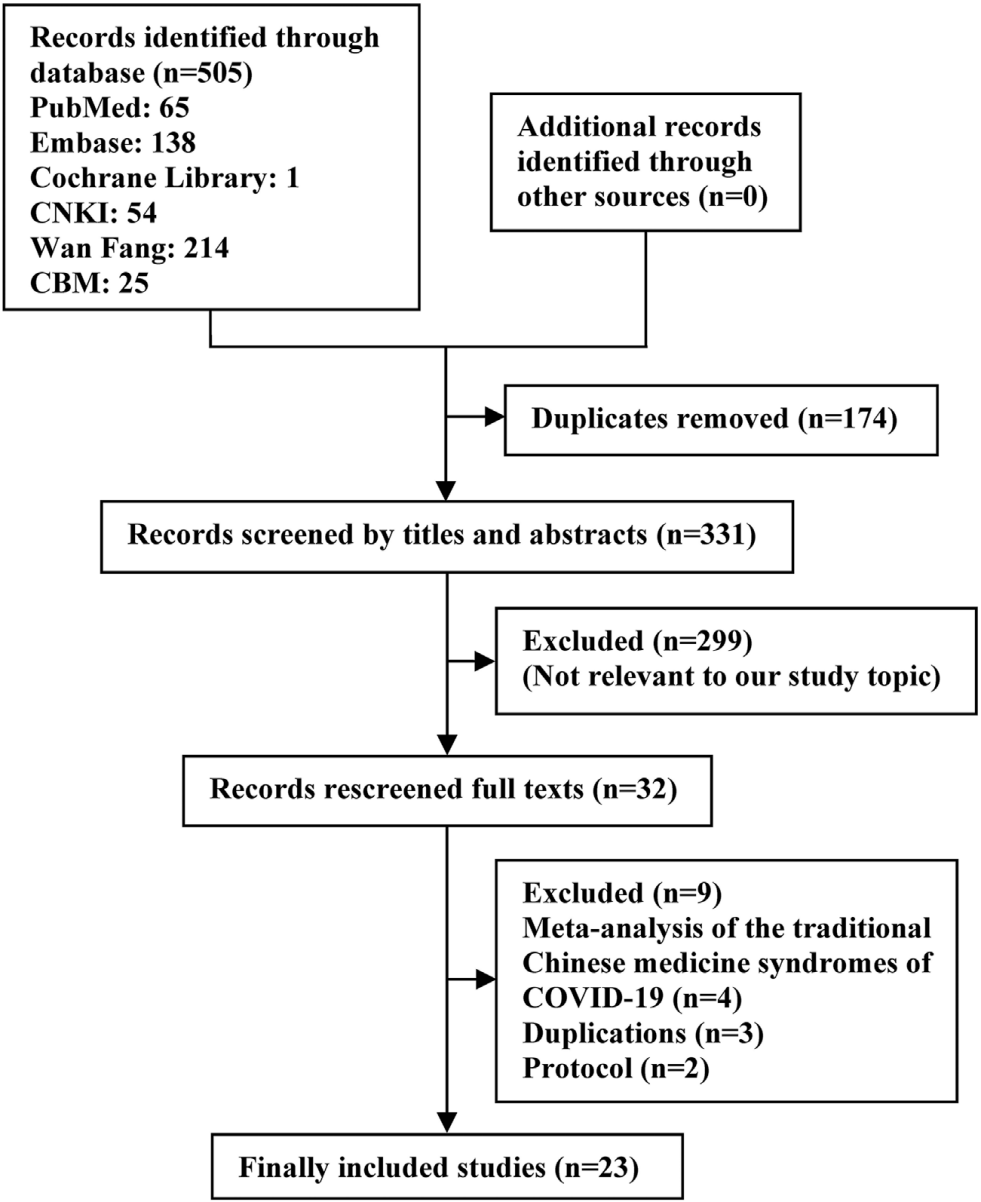
Flow diagram showing the search process and study selection.

A total of 505 studies were extracted from the electronic databases. After removing duplicates, 331 studies were screened by the titles and abstracts, and 32 studies were assessed through the full texts. Finally, 23 SRs were included in this study (Lin et al., 2020; Cai et al., 2020; Fan et al., 2020; Hu et al., 2020; Jin L. et al., 2020; Liu M. et al., 2021; Pang et al., 2020; Qi et al., 2020; Sun C. Y. et al., 2020; Xiong et al., 2020; Wang et al., 2020; Wu et al., 2020; Yang et al., 2020; Zhang W. B. et al., 2020; Zhang H. Y. et al., 2020; Gao et al., 2021; Liu et al., 2021; Luo et al., 2021; Liu A. H. et al., 2021; Ouyang et al., 2021; Shi et al., 2021; Zeng et al., 2020; Zhou et al., 2021). The literature screening procedure is shown in Figure 1.

### Study Characteristics

The basic characteristics of the included SRs are presented in Table 1. The 23 included SRs were published between 2020 and 2021, with 13 SRs in English (Liu et al., 2020; Cai et al., 2020; Fan et al., 2020; Hu et al., 2020; Jin L. et al., 2020; Liu M. et al., 2020; Pang et al., 2020; Sun C. Y. et al., 2020; Xiong et al., 2020; Luo et al., 2021; Shi et al., 2021; Zeng et al., 2020; Zhou et al., 2021) and 10 in Chinese (Qi et al., 2020; Wang et al., 2020; Wu et al., 2020; Yang et al., 2020; Gao et al., 2021; Liu A. H. et al., 2021; Ouyang et al., 2021; Zhang W. B. et al., 2020; Zhang H. Y. et al., 2020). The sample sizes ranged from 138 to 4,704 participants. In terms of the diagnostic criteria for COVID-19, five SRs used laboratory tests (Hu et al., 2020; Liu et al., 2020; Yang et al., 2020; Liu M. et al., 2021; Zhou et al., 2021), 11 SRs followed the New Coronavirus Pneumonia Diagnosis and Treatment Program (NCP-D and T program) (Jin L. et al., 2020; Pang et al., 2020; Qi et al., 2020; Wang et al., 2020; Wu et al., 2020; Zeng et al., 2020; Zhang W. B. et al., 2020; Zhang H. Y. et al., 2020; Luo et al., 2021; Ouyang et al., 2021; Liu A. H. et al., 2021), and seven SRs did not report the diagnostic methods used (Lin et al., 2020; Cai et al., 2020; Fan et al., 2020; Shi et al., 2021; Sun C. Y. et al., 2020; Xiong et al., 2020; Gao et al., 2021).

**TABLE 1:** General characteristics of the included systematic reviews (SRs)

Study ID	Type of disease	Diagnostic criteria	Included studies/patients (*n*)	Interventions	Control	Risk of bias tool	Data analysis methods	Outcomes
Xiong et al. (2020)	COVID-19	NR	18 (4,318)	(Qingfei Touxie Fuzheng recipe, Toujie Quwen granules, Jinhua Qinggan granules, Shufeng Jiedu capsule, Lianhua Qingwen granules, Lianhua Qingwen capsule) + WM	WM	Cochrane ROB	MA	(1), (6), (7), (10), (11), (13), (14), (18), (19), (21), (30), (34), (35), (36), (37), (40), (41), (42), (44), (45)
Liu et al. (2021)	COVID-19	Laboratory test	8 (924)	(Lianhua Qingwen granules, Lianhua Qingwen capsule) + WM	WM	Cochrane ROB; NOS	MA	(8), (1), (12)
Liu et al. (2021)	COVID-19	NR	7 (855)	(Lianhua Qingke granules, Shufeng Jiedu capsule, Jinhua Qinggan Granules, Toujie Quwen granules, Lianhua Qingwen granules) + WM	WM	Cochrane ROB	MA	(1), (3), (8), (16), (18), (19), (20), (21), (22), (23), (24), (27), (30), (35), (36), (38), (41), (45), (47), (48), (49)
Zhou et al. (2021)	COVID-19	Laboratory test	10 (1,285)	(Jinhua Qinggan granules, Qingfei Touxie Fuzheng recipe, Toujie Quwen granules, Lianhua Qingke granules, pneumonia no. 1 prescription granules, Jinyinhua oral liquid, Lianhua Qingwen capsule) + WM	WM	Cochrane ROB	MA	(1), (3), (7), (30), (34), (37)
Shi et al. (2021)	COVID-19	NR	48 (4,704)	Chinese herbal medicine + WM	WM	Cochrane ROB; NOS	MA	(12), (15), (28), (29), (59), (62), (64), (69)
Zeng et al. (2020)	COVID-19	NCP-D and T program (7th edition)	2 (154)	Lianhua Qingwen + WM	WM	Cochrane ROB	MA	(34), (38), (43), (47), (52), (54), (56), (57), (58), (60)
Liu et al. (2020)	COVID-19	Laboratory test	11 (982)	(Qingfei Touxie Fuzheng recipe, Shufeng Jiedu capsule, Lianhua Qingwen granules, Lianhua Qingwen capsule, Reyanning mixture, Toujie Quwen granules, Jinhua Qinggan granules) + WM	WM	Cochrane ROB; NOS	MA	(9), (7), (12), (13), (18), (19), (21), (25), (31), (32), (33), (34), (35), (38) (40), (43), (44), (52), (54), (56), (58), (63), (66), (67)
Sun et al. (2020)	COVID-19	NR	7 (681)	(Touxie Quwen recipe, Reyanning mixture, Shufeng Jiedu capsule, Qingfei Touxie Fuzheng recipe, pneumonia no. 1 prescription, Jinhua Qinggan granules) + WM	WM	Cochrane ROB	MA	(5), (9), (14), (18), (19), (20), (21), (26), (30)
Pang et al. (2020)	COVID-19	NCP-D and T program (5th–7th edition)	11 (1,259)	(Qingfei Touxie Fuzheng recipe, Jinhua Qinggan granules, Toujie Quwen granules, Lianhua Qingwen granules, Qingfei Paidu decoction, Maxing Xuanfei Jiedu decoction, Lianhua Qingke granules, Shufeng Jiedu capsule) + WM	WM	Cochrane ROB	MA	(11), (13), (14), (17), (30), (34), (35), (37), (40), (42), (44), (50), (62)
Luo et al. (2021)	COVID-19	NCP-D and T program	19 (1,474)	(Lianhua Qingwen granules, Shufeng Jiedu capsule, Toujie Quwen granules, Reyanning mixture, Jiawei Dayuan decoction, pneumonia no. 1 prescription, Jinhua Qinggan granules, Qingfei Paidu decoction, Xuebijing, Qingfei Touxie Fuzheng recipe) + WM	WM	Cochrane ROB	MA	(1), (8), (11), (13), (14), (30), (34), (38), (43)
Jin L. et al. (2020)	COVID-19	NCP-D and T program (6th edition)	5 (598)	(Qingfei Touxie Fuzheng recipe, Lianhua Qingwen granules, Lianhua Qingke granules, Xuebijing injection) + WM	WM	Jadad scale	MA	(9)
Hu et al. (2020)	COVID-19	laboratory tests	7 (942)	(Lianhua Qingwen granules, Lianhua Qingwen capsule) + WM	WM	Jadad scale	MA	(2), (7), (11), (13), (34), (37), (42), (46), (51), (53), (55), (59), (60), (62), (68)
Fan et al. (2020)	COVID-19	NR	7 (732)	(Qingfei Touxie Fuzheng recipe, Jinhua Qinggan granules, Toujie Quwen granules, Lianhua Qingwen capsule, Maxing Shigan–Dayuanyin decoction, Jiawei Dayuan granules) + WM	WM	Cochrane ROB	MA	(1), (21)
Ouyang et al. (2021)	Mild or common COVID-19	NCP-D and T program	10 (832)	(Reyanning mixture, Jinhua Qinggan granules, Toujie Quwen granules, Shufeng Jiedu capsule, Lianhua Qingwen capsule) + WM	WM	Cochrane ROB; NOS	MA	(4), (8), (12), (14), (18), (19), (20), (30), (34), (35), (38), (43)
Liu et al. (2021)	COVID-19	NCP-D and T program (7th edition)	7 (682)	(Lianhua Qingwen granules, Lianhua Qingwen capsule, Jinhua Qinggan granules, Shufeng Jiedu capsule, Xuebijing injection) + WM	WM	Jadad Scale; NOS	MA	(1), (8), (11), (34), (35), (38), (43), (47)
Gao et al. (2021)	COVID-19	NR	12 (912)	(Pneumonia no. 1 prescription, Qingfei Touxie Fuzheng recipe, Shufeng Jiedu capsule, Lianhua Qingwen granules, Jinhua Qinggan granules, Touxie Quwen granules, Reyanning mixture) + WM	WM	Cochrane ROB; NOS	MA	(1), (8), (11), (14), (34), (35), (36), (37), (39), (41), (42), (44), (45)
Zhang et al. (2020)	Common COVID-19	NCP-D and T program	5 (600)	(Lianhua Qingwen granules, Lianhua Qingwen capsule) + WM	WM	Cochrane ROB	MA	(9), (30), (34), (35), (38), (43), (54)
Zhang et al. (2020)	COVID-19	NCP-D and T program	5 (597)	(Lianhua Qingwen granules, Lianhua Qingwen capsule) + WM	WM	Cochrane ROB	MA	(11), (34), (38), (43)
Yang et al. (2020)	COVID-19	Laboratory test	3 (138)	(Lianhua Qingwen granules, Lianhua Qingwen capsule) + WM	WM	NR	MA	(34), (38), (43), (56), (57), (61)
Wu et al. (2020)	COVID-19	NCP-D and T program (5th edition)	8 (804)	(Qingfei Touxie Fuzheng recipe, Lianhua Qingwen granules, Shufeng Jiedu granules) + WM	WM	Cochrane ROB; NOS	MA	(4), (6), (7), (11), (35), (37), (62)
Wang et al. (2020)	COVID-19	NCP-D and T program	7 (665)	(Lianhua Qingwen granules, Lianhua Qingwen capsule, Shuanghuanglian oral liquid, Yupingfeng granules, Shiduyufei decoction) + WM	WM	Cochrane ROB	MA	(1), (11), (13), (35)
Qi et al. (2020)	Common COVID-19	NCP-D and T program	4 (181)	Lianhua Qingwen granules + WM	WM	Cochrane ROB	MA	(9), (34), (38), (43), (47), (56), (61)
Cai et al. (2020)	COVID-19	NR	6 (463)	(Toujie Quwen granules, Jinhua Qinggan granules, Lianhua Qingwen granules, Shufeng Jiedu capsule) + WM	WM	Cochrane ROB; NOS	MA	(1), (7), (8), (11), (30), (34), (35), (38), (39), (43), (44)

*NCP-D and T program*, New Coronavirus Pneumonia Diagnosis and Treatment program; *SRs*, systematic reviews; *MA*, meta-analysis; *Cochrane ROB*, Cochrane risk of bias tool; *NOS*, Newcastle–Ottawa Scale; *NR*, not reported; *WM*, Western medicine (includes antibacterial, antiviral, hormone therapy, respiratory support, etc., conventional medications)

Outcomes: (*1*) CT recovery rate. (*2*) Improvement rate of pulmonary imaging. (*3*) Absorption rate of lesions. (*4*) Absorption rate of pneumonia. (*5*) Remission rate of pulmonary inflammation. (*6*) Mortality. (*7*) Cure rate. (*8*) Total effective rate. (*9*) Clinical efficacy. (*10*) Rate of range from critical to mild cases. (*11*) Rate of range from mild to critical cases. (*12*) Aggravation of COVID-19. (*13*) Hospital stay. (*14*) Viral nucleic acid negative conversion rate. (*15*) Time to viral assay conversion. (*16*) Hospital discharge rate. (*17*) All-cause death. (*18*) White blood cell counts. (*19*) Lymphocyte counts. (*20*) Lymphocyte percentage. (*21*) Amount of C-reactive protein. (*22*) Total procalcitonin level. (*23*) Neutrophil percentage. (*24*) d-Dimer level. (*25*) TNF-α level. (*26*) IL-6 level. (*27*) Oxygenation index. (*28*) Effective rate of ALT returning to normal. (*29*) Effective rate of AST returning to normal. (*30*) Adverse effects. (*31*) Rate of diarrhea. (*32*) Rate of nausea and vomiting. (*33*) Rate of liver damage. (*34*) Disappearance rate of fever. (*35*) Disappearance time of fever. (*36*) Symptom score of fever. (*37*) Rate of cough reduction. (*38*) Cough disappearance rate. (*39*) Cough disappearance time. (*40*) Cough reduction time. (*41*) Symptom score of cough. (*42*) Rate of fatigue reduction. (*43*) Disappearance rate of fatigue. (*44*) Disappearance time of fatigue. (*45*) Symptom score of fatigue. (*46*) Improvement rate of sputum. (*47*) Disappearance rate of sputum. (*48*) TCM syndrome score of dry and sore throat. (*49*) Rate of anxiety relief. (*50*) Body pain resolution rate. (*51*) Improvement rate of muscle pain. (*52*) Disappearance rate of muscle pain. (*53*) Improvement rate of shortness of breath. (*54*) Disappearance rate of shortness of breath. (*55*) Improvement rate of chest tightness. (*56*) Disappearance rate of chest tightness. (*57*) Disappearance rate of difficulty breathing. (*58*) Disappearance rate of nausea. (*59*) Improvement rate of nausea. (*60*) Improvement rate of loss of appetite. (*61*) Disappearance rate of loss of appetite. (*62*) Improvement rate of diarrhea. (*63*) Disappearance rate of diarrhea. (*64*) Remission rate of anorexia. (*65*) Anorexia disappearance rate. (*66*) Nasal congestion disappearance time. (*67*) Runny nose disappearance time. (*68*) Rate of improvement sore throat. (*69*) Remission rate of vomiting

Out of the eligible SRs, seven included Lianhua Qingwen granules/capsules + WM as interventions (Hu et al., 2020; Qi et al., 2020; Yang et al., 2020; Zeng et al., 2020; Zhang W. B. et al., 2020; Zhang H. Y. et al., 2020; Liu M. et al., 2021). Sixteen SRs reported TCM + WM as an intervention, in which TCM interventions included herbal decoctions (Qingfei Touxie Fuzheng recipe, Qingfei Paidu decoction, Maxing Xuanfei Jiedu decoction, etc.) and Chinese patent medicine (Lianhua Qingwen granules/capsule, Shufeng Jiedu capsule, Toujie Quwen granules, etc.) (Ang et al., 2020; Cai et al., 2020; Fan et al., 2020; Jin et al., 2020; Liu et al., 2020; Pang et al., 2020; Sun C. Y. et al., 2020; Wang et al., 2020; Wu et al., 2020; Xiong et al., 2020; Gao et al., 2021; Liu A. H. et al., 2021; Luo et al., 2021; Ouyang et al., 2021; Shi et al., 2021; Zhou et al., 2021). The name and composition of each TCM reported in original studies of the included SRs are given in *Supplementary Material S3*.

The Cochrane risk of bias tool was used in 12 SRs (Fan et al., 2020; Lin et al., 2020; Pang et al., 2020; Sun et al., 2020; Qi et al., 2020; Wang et al., 2020; Xiong et al., 2020; Zeng et al., 2020; Zhang et al., 2020; Zhang et al., 2020; Luo et al., 2021; Zhou et al., 2021), while the Jadad scale was referred to in two SRs (Hu et al., 2020; Jin et al., 2020). Seven SRs used the Cochrane risk of bias tool and the Newcastle–Ottawa Scale (NOS) (Cai et al., 2020; Liu et al., 2020; Wu et al., 2020; Gao et al., 2021; Liu M. et al., 2021; Ouyang et al., 2021; Shi et al., 2021), while one SR used the Jadad Scale and NOS (Liu A. H. et al., 2021). One SR did not report the tool used in methodological assessment (Yang et al., 2020).

### Methodological Quality of the Included SRs

**FIGURE 2:**
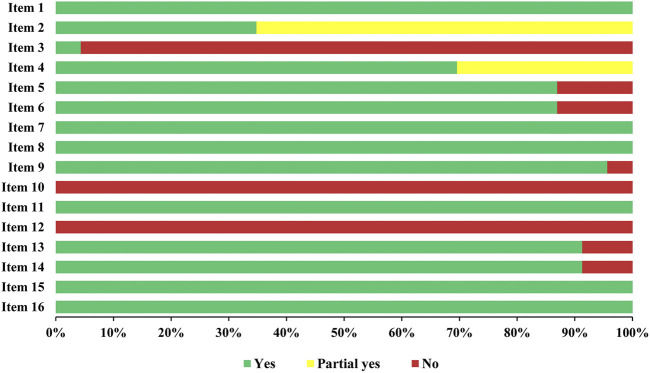
Methodological quality of the included systematic reviews (SRs).

The results of the AMSTAR 2 assessment are shown in Figure 2. For each AMSTAR 2 item, among the 16 items, six items were rated as “Yes” (items 1, 7, 8, 11, 15, and 16). Eight SRs (34.8%) reported the predefined protocol (item 2), 1 SR (4.3%) provided the reason for including the studies (item 3), 16 SRs (70%) provided the comprehensive search strategy along with supplementary search (item 4), 20 SRs (87%) conducted study selection and data extraction (items 5 and 6) using two reviewers, 22 SRs (95.7%) provided appropriate risk of bias tools for the reviews (item 9), no SR provided the sources of funding for the studies included in the review (item 10) and assessed the potential impact of risk of bias in individual studies (item 12), and 22 SRs (91.3%) accounted for risk of bias in individual studies when interpreting the results (item 13) and provided a satisfactory explanation for the heterogeneity in the results of the review (item 14). For overall methodological quality, 21 SRs were rated as moderate confidence, 1 SR was rated as low confidence, and 1 SR was rated as very low confidence. Detailed information related to the results of the AMSTAR 2 assessment is shown in *Supplementary Material S4*. The differences in the AMSTAR 2 assessment results between the included SRs published in English and those in Chinese are shown in *Supplementary Material S5*.

### Risk of Bias of the Included SRs

**FIGURE 3:**
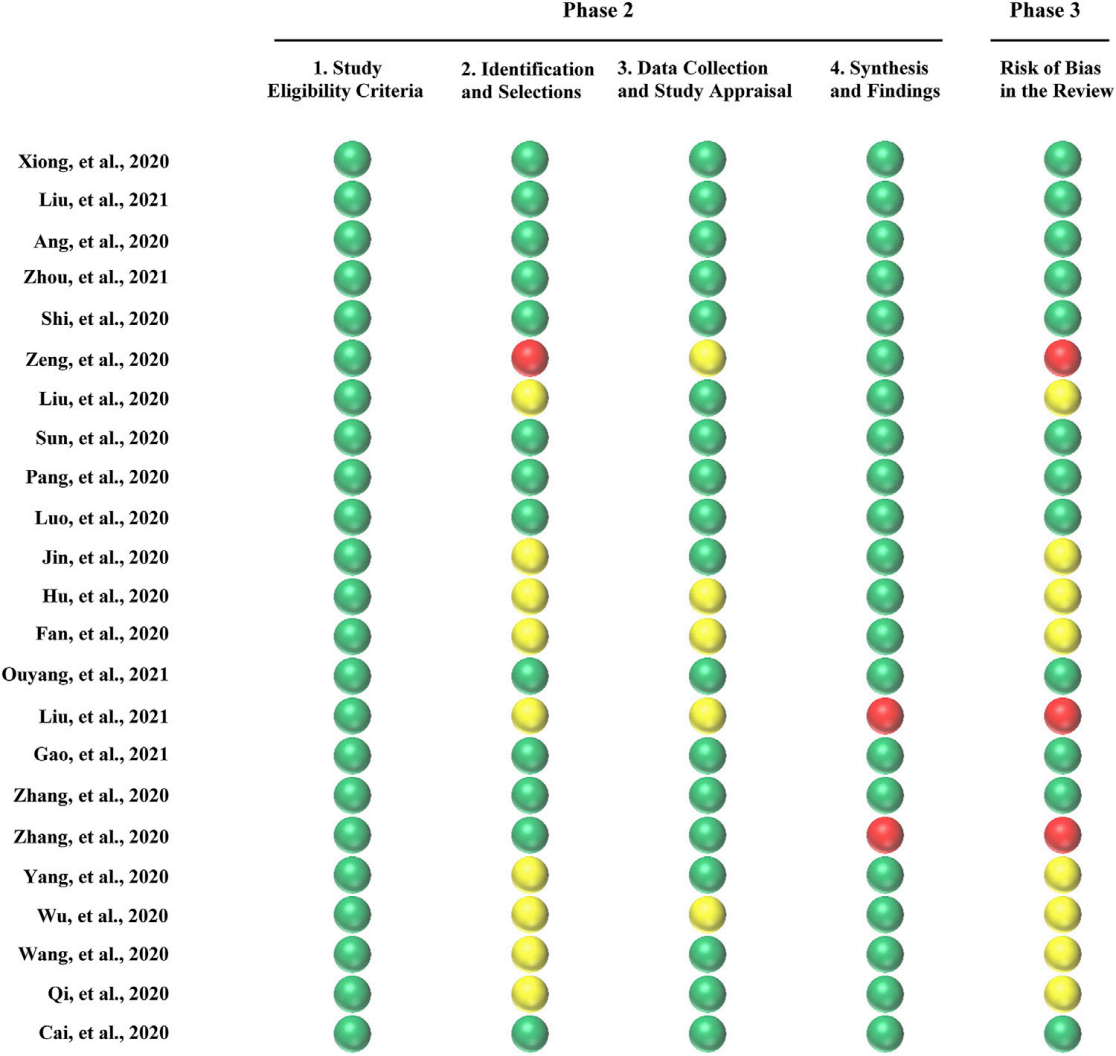
Mapping of risk of bias of the included systematic reviews (SRs). Green color represented low risk bias, and red color represented high risk bias, and yellow color represented unclear risk bias. The ROBIS tool items were represented on the lateral axis and the included SRs was represented on the vertical axis.

The results of risk of bias of the included SRs are shown in Figure 3. The ROBIS tool includes three phases with four domains. All SRs conformed to the target question; hence, phase 1 was rated as “Yes.” Phase 2 has four domains. For domain 1, which assesses concerns regarding the specification of the study eligibility criteria, 23 SRs (100%) were rated as at low risk. Domain 2 assesses the identification and selection, in which 13 SRs (57%) were rated as at low risk, nine SRs (39%) were rated as at unclear risk, and one SR (4%) was rated as at high risk because only one reviewer carried out the selection of studies. With regard to domain 3—data collection and study appraisal—18 SRs (78%) were rated as at low risk and five SRs (22%) were rated as at unclear risk. For domain 4—synthesis and findings—21 SRs (91%) were rated as at low risk and two SRs (8%) were rated as at high risk. Phase 3 considers the overall risk of bias of the 23 included SRs, with 12 SRs (52%) rated as at low risk, 8 SRs (35%) rated as at unclear risk, and 3 SRs (13%) rated as at high risk.

### Summary of the Risk of Bias of RCTs and OSs Included in the SRs

**FIGURE 4:**
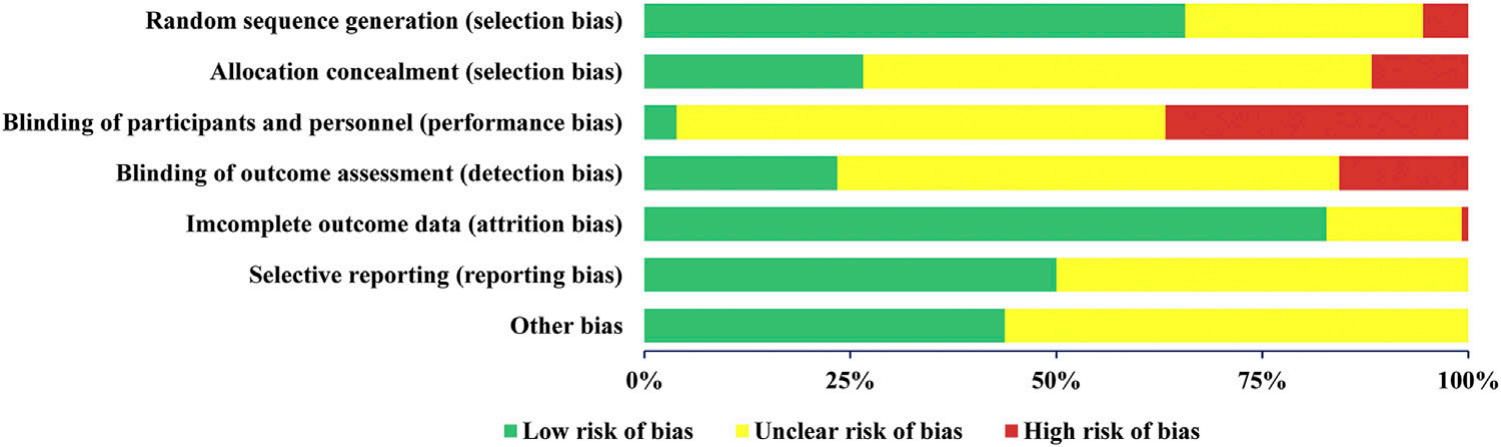
Summary of the risk of bias of the randomized controlled trials (RCTs) included in the systematic reviews (SRs).

The summary of risk of bias of RCTs included in the SRs, as assessed by the Cochrane risk of bias tool, is given in Figure 4. Eighty-four RCTs (66%) reported adequate random sequence generation, while 34 (27%) described an appropriate method of allocation concealment. Only 5 studies (4%) blinded the participants and personnel, while 30 RCTs (23%) used blinded outcome assessors. The risk of bias for incomplete outcome data was low for 106 RCTs (83%). Selective outcome reporting was regarded as low risk in 64 RCTs (50%). The risks of other biases were considered as low risk for 56 RCTs (44%). Furthermore, as per the summary of risk of biases of OSs included in the SRs, scored with the NOS, 10 OSs (12%) were rated with 8 points, 38 (47%) with 7 points, 15 (19%) with 6 points, 7 (8.5%) with 5 points, 7 (8.5%) with 4 points, and 4 OSs (5%) with 3 points.

### Evidence Quality of Outcomes

The quality of evidence for every quantitative synthesis outcome of the 23 included SRs was assessed using the GRADE approach and categorized as “low,” “moderate,” and “high.” Overall, most of the outcomes were rated as having moderate quality. For the RCTs, the quality of evidence was downgraded because of the following limitations: 1) the methodological quality of the RCTs included in individual SRs was poor; 2) inconsistency was present mainly because the heterogeneity test *p*-value was very small and the *I* ^2^ value was large; and 3) most of the findings were found to have a low precision because of the suboptimal sample size and wide confidence intervals (CIs). A larger effect was the most common upgrading factor in the OSs. Details of evidence quality for each outcome are given in *Supplementary Material S6*.

### Efficacy Outcomes

**FIGURE 5:**
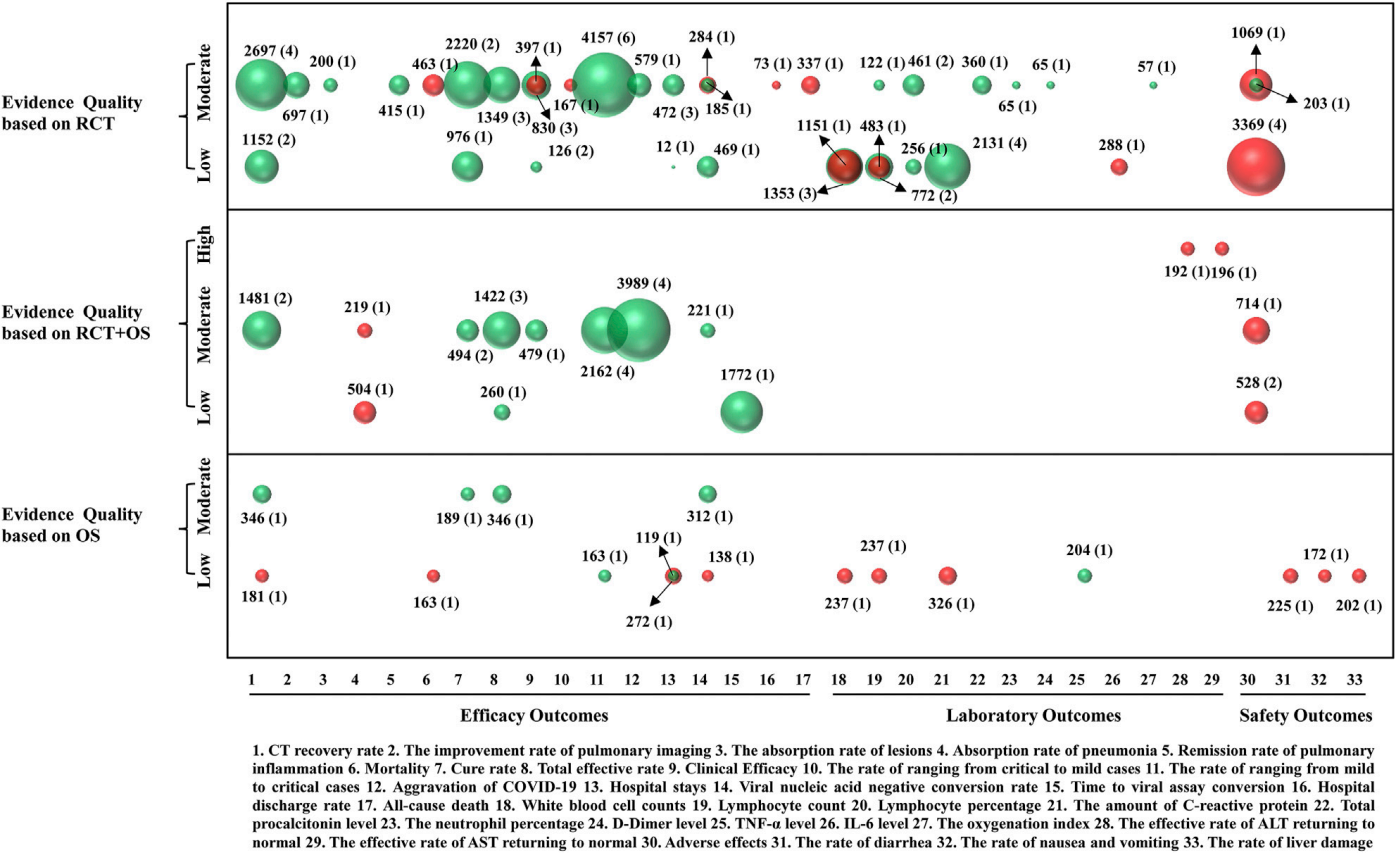
Mapping of evidence quality of efficacy, laboratory, and safety outcomes. Each bubble size represented patients included in the SRs, green color represented *p*<0.05, and red color represented *p*>0.05. The outcomes were represented on the lateral axis, and the evidence quality of outcome was represented on the vertical axis.

There were 17 efficacy outcomes in TCM + WM compared with WM studies (Figure 5). The CT recovery rate was reported in 10 SRs (43%), and the levels of evidence were moderate quality in seven (four SRs of RCTs, two SRs of RCTs and OSs, and one SR of OSs) and low quality in three (two SRs of RCTs and one SR of OSs). The improvement rate of pulmonary imaging, absorption rate of lesions (chest CT), remission rate of pulmonary inflammation, conversion rate from mild to critical, the hospital discharge rate, and the all-cause death rate were each reported in one SR of RCTs (4%), with moderate-quality evidence. The absorption rate of pneumonia was reported in two SRs of RCTs and OSs (9%), one with moderate-quality and one with low-quality evidence.

The cure rate was reported in six SRs (26%), five with moderate-quality evidence (two SRs of RCTs, two SRs of RCTs and OSs, and one SR of OSs) and one low-quality evidence (SR of OSs). The total effective rate was reported in eight SRs (35%), and the levels of evidence were moderate quality in seven (three SRs of RCTs, three SRs of RCTs and OSs, and one SR of OSs) and low quality in one (SR of RCTs and OSs). Clinical efficacy was reported in four SRs (17%), and the levels of evidence were moderate quality in three (two SRs of RCTs and one SR of RCTs and OSs) and low quality in one (SR of RCTs). The rate of transition from mild to critical was reported in 11 SRs (48%), with 10 having moderate-quality evidence (six SRs of RCTs and four SRs of RCTs and OSs) and one with low-quality evidence (SR of OSs).

The aggravation rate of COVID-19 was reported in four SRs (17%), all with moderate-quality evidence (one SR of RCTs and three SRs of RCTs and OSs). The length of hospital stay was reported in six SRs (26%), and the levels of evidence were moderate quality in three (SRs of RCTs) and low quality in three (one SR of RCTs and two SRs of OSs). Mortality was reported in two SRs (9%), one with moderate-quality evidence (SR of RCTs) and one with low-quality evidence (SR of OSs). The viral nucleic acid negative conversion rate was reported in six SRs (26%), and the levels of evidence were moderate quality in four (two SRs of RCTs, one SR of RCTs and OSs, and one SR of OSs) and low quality in two (one SR of RCTs and one SR of OSs). Time to viral assay conversion was reported in one SR of RCTs and OSs (4%), with low-quality evidence.

The results showed that, compared with WM approaches, TCM + WM approaches could improve the CT recovery rate, improvement rate of pulmonary imaging, absorption rate of lesions, remission rate of pulmonary inflammation, cure rate, total effective rate, clinical efficacy, and the viral nucleic acid negative conversion rate (*p* < 0.05). These approaches could decrease the aggravation of COVID-19 and the rate of transition from mild to critical (*p* < 0.05) and also shorten the time to viral assay conversion and length of hospital stay (*p* < 0.05).

### Laboratory Outcomes

There were 12 laboratory outcomes in TCM + WM and WM studies (Figure 5). White blood cell counts were reported in five SRs (22%), with all five showing low-quality evidence (four SRs of RCTs and one SR of OSs). Lymphocyte counts were reported in five SRs (22%), and the levels of evidence were moderate quality in one (SR of RCTs) and low quality in four (three SRs of RCTs and one SR of OSs). Lymphocyte percentage was reported in three SRs of RCTs (13%), and the levels of evidence were moderate quality two and low quality in one. The amount of C-reactive protein was reported in five SRs (22%), all with low-quality evidence (four SRs of RCTs and one SR of OSs).

The total procalcitonin level, neutrophil percentage, and the d-dimer level were each reported in one SR of RCTs (4%), with moderate-quality evidence. The tumor necrosis factor α (TNF-α) level was reported in one SR of OSs (4%), with low-quality evidence, while the interleukin 6 (IL-6) level was reported in one SR of RCTs (4%), with low-quality evidence. The oxygenation index was reported in one SR of RCTs (4%), with moderate-quality evidence, while the effective rates of alanine transaminase (ALT) and aspartate transaminase (AST) returning to normal were each reported in one SR of RCTs and OSs (4%), with high-quality evidence.

Compared to WM approaches, TCM + WM approaches could significantly reduce white blood cell counts, lymphocyte counts, C-reactive protein, total procalcitonin level, neutrophil percentage, the d-dimer level, and the TNF- level and also improve the lymphocyte percentage and the oxygenation index (*p* < 0.05).

### Safety Outcomes

There were four safety outcomes in studies on TCM + WM and WM approaches (Figure 5). Adverse events were reported in nine SRs (39%), and their levels of evidence were moderate quality in three (two SRs of RCTs and one SR of RCTs and OSs) and low quality in six (four SRs of RCTs and two SRs of RCTs and OSs). The rate of diarrhea, rate of nausea with vomiting, and liver damage were each reported by one SR of OSs (4%), with low-quality evidence.

The results showed that TCM + WM approaches could reduce adverse events, rate of nausea with vomiting, and liver damage when compared to the WM approaches (*p* > 0.05).

### Clinical Symptom Outcomes

**FIGURE 6:**
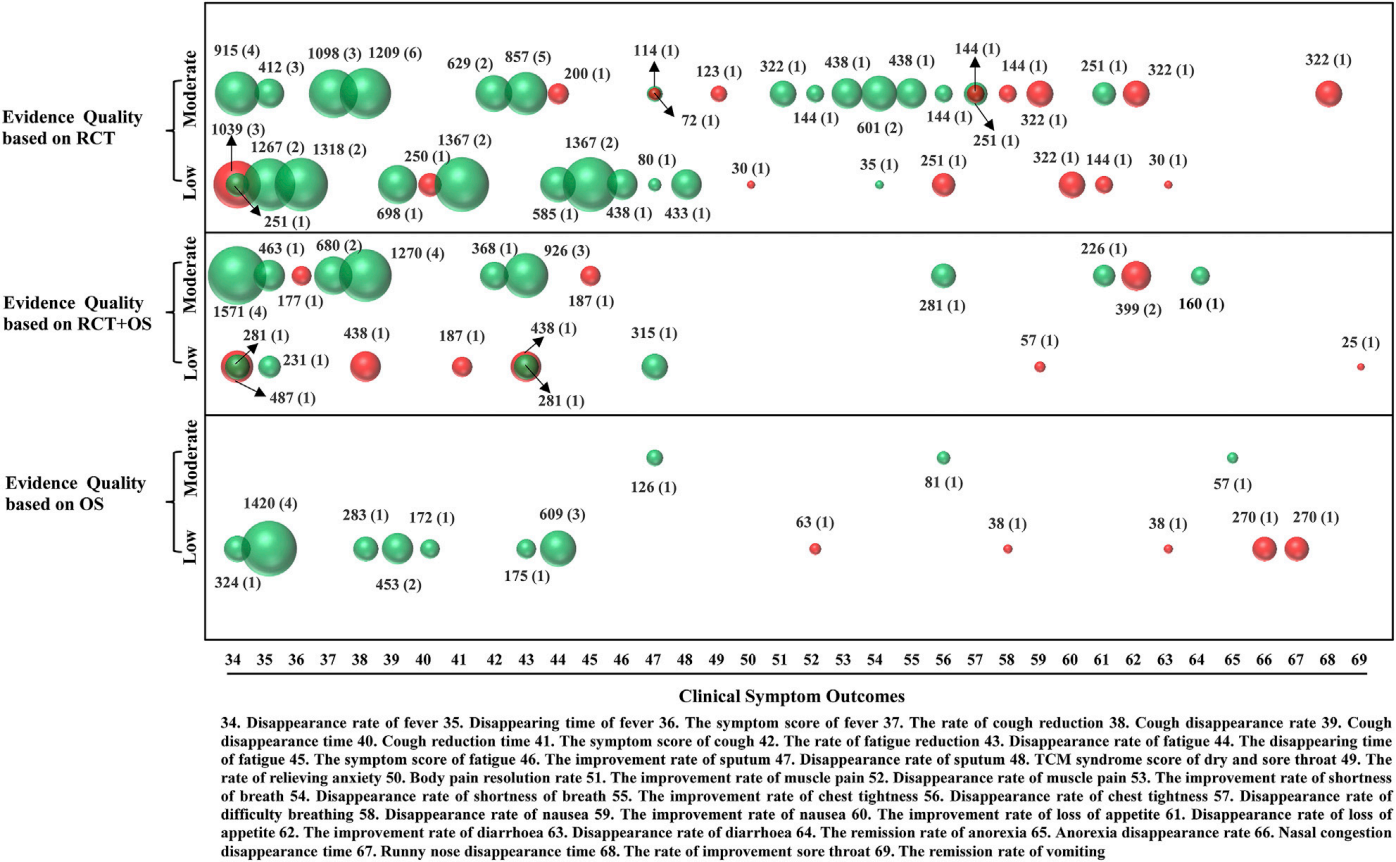
Mapping of evidence quality of clinical symptom outcomes. Each bubble size represents patients included in the SRs, green color represented *p*<0.05, and red color represented *p*>0.05. The outcomes were represented on the lateral axis, whereas the evidence quality was represented on the vertical axis.

There were 36 clinical symptom outcomes in the TCM + WM and WM studies (Figure 6). The disappearance rate of fever was reported in 15 SRs (65%), and the levels of evidence were moderate quality in seven (three SRs of RCTs and four SRs of RCTs and OSs) and low quality in eight (five SRs of RCTs, two SRs of RCTs and OSs, and one SR of OSs). The disappearance time of fever was reported in 11 SRs (48%), and the levels of evidence were moderate quality in four (three SRs of RCTs and one SR of RCTs and OSs) and low quality in seven (two SRs of RCTs, one SR of RCTs and OSs, and four SRs of OSs). The symptom score of fever was reported in three SRs (13%), one with moderate-quality (SR of RCTs and OSs) and two with low-quality (SRs of RCTs) evidence.

The rate of cough reduction was reported in five SRs (22%), all with moderate-quality evidence (three SRs of RCTs and two SRs of RCTs and OSs). The cough disappearance rate was reported in 12 SRs (52%), and the levels of evidence were moderate quality in 10 (six SRs of RCTs and four SRs of RCTs and OSs) and low quality in two (one SR of RCTs and OSs and one SR of OSs). The cough reduction time was reported in two SRs (9%), both with low-quality evidence (one SR of RCTs and one SR of OSs). The cough disappearance time was reported in three SRs (13%), all three with low-quality evidence (one SR of RCTs and two SRs of OSs). The symptom score of cough was reported in three SRs (13%), all three with low-quality evidence (two SRs of RCTs and one SR of OSs).

The rate of fatigue reduction was reported in three SRs (13%), all with moderate-quality evidence (two SRs of RCTs and one SR of RCTs and OSs). The disappearance rate of fatigue was reported in 11 SRs (49%), and their levels of evidence were moderate quality in eight (five SRs of RCTs and three SRs of RCTs and OSs) and low quality in three (two SRs of RCTs and OSs and one SR of OSs). The disappearance time of fatigue was reported in five SRs (22%), one with moderate-quality evidence (one SR of RCTs) and four with low-quality evidence (one SR of RCTs and three SRs of OSs). The symptom score of fatigue was reported in three SRs (13%), and the levels of evidence were moderate quality in one (SR of RCTs and OSs) and low quality in two (SRs of RCTs).

The improvement rate of sputum was reported in one SR of RCTs (4%), with moderate-quality evidence. The disappearance rate of sputum was reported in five SRs (22%), and the levels of evidence were moderate quality in three (two SRs of RCTs and one SR of OSs) and low quality in two (one SR of RCTs and one SR of RCTs and OSs).

The rate of sore throat improvement was reported in one SR of RCTs (4%), with moderate-quality evidence. The TCM syndrome score of dry and sore throat was reported in one SR of RCTs (4%), with low-quality evidence. The disappearance times of nasal congestion and runny nose were each reported in one SR of OSs (4%), with low-quality evidence, while the body pain resolution rate was reported in one SR of RCTs (4%), with low-quality evidence. The improvement rate of muscle pain was reported in one SR of RCTs (4%), with moderate-quality evidence, and the disappearance rate of muscle pain was reported in two SRs (9%), one with moderate-quality evidence (SR of RCTs) and one with low-quality evidence (SR of OSs).

The rates of improvement of shortness of breath and chest tightness were reported in one SR of RCTs (4%), with moderate-quality evidence. The disappearance rate of shortness of breath was reported in three SRs (13%), two with moderate-quality evidence (SRs of RCTs) and one with low-quality evidence (SR of RCTs). The disappearance rate of chest tightness was reported in four SRs (17%), and their levels of evidence were moderate quality in three (one SR of RCTs, one SR of RCTs and OSs, and one SR of OSs) and low quality in one (SR of RCTs). The disappearance rate of difficulty in breathing was reported in two SRs of RCTs (9%), both with moderate-quality evidence.

The disappearance rate of nausea was reported in two SRs (9%), one with moderate-quality evidence (SR of RCTs) and one with low-quality evidence (SR of OSs), while the improvement rate of nausea was reported in two SRs (9%), one with moderate-quality evidence (SR of RCTs) and one with low-quality evidence (SR of RCTs and OSs). The remission rate of vomiting was reported in one SR of RCTs and OSs (4%), with low-quality evidence. The improvement rate of loss of appetite was reported in one SR of RCTs (4%), with low-quality evidence, and the disappearance rate of loss of appetite was reported in three SRs (13%), two with moderate-quality evidence (one SR of RCTs and one SR of RCTs and OSs) and one with low-quality evidence (SR of RCTs). The improvement rate of diarrhea was reported in three SRs (13%), all with moderate-quality evidence (one SR of RCTs and two SRs of RCTs and OSs). The disappearance rate of diarrhea was reported in two SRs (9%), both with low-quality evidence (one SR of RCTs and one SR of OSs). The remission rate of anorexia was reported in one SR of RCTs and OSs (4%), with moderate-quality evidence, while the anorexia disappearance rate was reported in one SR of OSs (4%), with moderate-quality evidence. The rate of relieving anxiety was reported in one SR of RCTs (4%), with moderate-quality evidence.

Compared with WM approaches, TCM + WM approaches could improve the disappearance rate of fever, cough, fatigue, sputum, muscle pain, shortness of breath, chest tightness, loss of appetite, and anorexia (*p* < 0.05) and enhance the remission rate of cough, fatigue, sputum, muscle pain, shortness of breath, chest tightness, and anorexia (*p* < 0.05). These approaches could further shorten the disappearance time of fever, cough, and fatigue and cut down the cough reduction time compared to WM (*p* < 0.05). TCM + WM approaches could also decrease the symptom score of fever, cough, fatigue, and dry and sore throat compared with WM approaches (*p* < 0.05).

## Discussion

### Summary of the Main Findings

Twenty-three SRs were included in this study. We critically assessed the included SRs using AMSTAR 2, ROBIS tool, and the GRADE system. Using AMSTAR 2, we categorized 21 SRs as having moderate confidence, one SR of low confidence, and one SR of very low confidence. Using the ROBIS tool, we rated 12 SRs at low risk, eight at unclear risk, and three at high risk. With the GRADE system, most outcomes were graded as having moderate quality. The results suggested that TCM + WM approaches could improve the efficacy, laboratory, and clinical symptom outcomes compared with WM approaches in COVID-19 patients.

### Inflammatory Mechanisms Associated With TCM in COVID-19 Patients

COVID-19 is caused by SARS-CoV-2, with basic reproduction number (R0) of person-to-person transmission of 2.6, meaning that infected cases of SARS-CoV-2 increase at an exponential rate (Li et al., 2020). When the respiratory tract is infected by pathogens, the host immune system is activated to resist and clear the infection. Airway epithelium cells and alveolar macrophages release multiple pro-inflammatory cytokines and chemokines, such as TNF-α, IL-6, interferons (IFNs), colony-stimulating factors (CSFs), interleukin 8 (IL-8), monocyte chemoattractant protein-1 (MCP-1), and macrophage inflammatory protein (MIP). This robust cytokine production is known as the “cytokine storm” (Zhang et al., 2013; Ding et al., 2017). Huang et al. validated this excessive release of inflammatory cytokines (cytokine storm), including IL-1β, IL-6, IL-12, IFN-γ, IP-10, and MCP-1, in COVID-19 patients admitted to the intensive care unit (Huang et al., 2020). Run et al. reported that host cells infected with SARS-CoV-2 released cytokines such as TNF-α, IL-6, MCP-1, and CXCL-10 (C-X-C motif chemokine, CXCL), and the elevated expressions of these four cytokines were significantly inhibited by Lianhua Qingwen in a concentration-dependent manner (Huang et al., 2020). Additionally, the study of Ding et al. showed that the Qingfei Touxie Fuzheng recipe significantly decreased the levels of IL-6 compared with WM (*p* < 0.05) (Ding et al., 2020).

### Methodological Quality of the Included SRs

There were several defects in the methodological quality of the included SRs. Based on AMSTAR 2, one SR was rated as low confidence since the the risk of bias in individual studies when discussing the results of the SR was not accounted for (Liu A. H. et al., 2021). Another SR was rated as very low confidence because the risk of bias for the included studies was not assessed (Yang et al., 2020). In addition, item 3 (“explain their selection of the study designs for inclusion in the review”), item 10 (“reporting on the sources of funding for the studies included in the review”), and item 12 (“assess the potential impact of RoB in individual studies on the results of the meta-analysis”) also needed improvement. All of the above-mentioned limitations affected the confidence of the included SRs.

### Risk of Bias of the Included SRs

Several limitations related to the risk of bias of the included SRs should have been addressed. Using the ROBIS tool, we found that there were some risks of bias in domains 2–4 of phase 2. In domain 2, we focused on the risk of bias in the identification and selection of studies. The results indicated that reviewers of the SRs did not pay attention to whether the search included additional search methods, such as citation searches, contacting experts, reference checking, or manual search, which may reduce publication bias and contribute to a comprehensive evaluation of evidence. Additionally, to reduce random errors, two researchers should have conducted the selection of studies, independently. For domain 3 of data collection and study appraisal, the risk of bias in five SRs (Fan et al., 2020; Hu et al., 2020; Wu et al., 2020; Liu A. H. et al., 2021; Ouyang et al., 2021) was unclear because duplicate data extraction was not reported, which was needed to safeguard against random errors. In domain 4, the risk of bias in two SRs (Liu et al., 2021; Zhang et al., 2020) was high because the high risk of original studies in their results and discussion sections was not interpreted appropriately.

### Risk of Bias of the RCTs and OSs Included in the SRs

Regarding the risk of bias of the RCTs included in SRs, poor methodological design was a very common problem in most studies. These drawbacks included sequence generation of randomization, concealment of allocation, and reporting on blinding. With regard to the risk of bias of the OSs included in the SRs, 11 OSs were judged as having unsatisfactory quality, mainly due to low scores in population selection, comparability between the exposed and unexposed groups, and adequacy of follow-up. Future observational studies should pay attention to these three aspects of methodological design.

### Evidence Quality of Outcomes

In the assessment of evidence quality of outcomes using the GRADE system, it was found that the quality of evidence for outcomes was low, moderate, and high, and the majority of outcomes were graded as having moderate-quality evidence. Of the five downgrading factors, the risk of bias was the most common factor downgrading the level of evidence. The main methodological drawbacks included the following: RCTs missed the method of random sequence generation; most RCTs did not state that the treatment allocation was concealed; and most RCTs did not show whether they blinded the practitioners and patients. Consequently, clinical researchers should pay more attention to the details of the research design, which would result in high-quality RCTs in the future. Inconsistency was the second downgrading factor, owing to the high *I* ^2^ value and statistically significant heterogeneity of the effect estimates. The GRADE system suggested that the authors of SRs should generate and test a small number of *a priori* hypotheses related to patients, interventions, outcomes, and methodology to explore the sources of heterogeneity and resolve inconsistency (Guyatt et al., 2011). Inaccuracy was the third downgrading factor, due to the insufficient sample size and wide 95% CIs, which indicates that multicenter, large-sample RCTs are needed in the future.

### Strengths and Limitations

This study has several strengths. Firstly, we performed a comprehensive search to identify SRs relating to TCM + WM approaches for COVID-19 from six databases. Secondly, we assessed the methodological quality and the risk of bias of the included SRs using AMSTAR 2 and ROBIS tool and also assessed the evidence quality of outcomes of the included SRs with the GRADE system. Thirdly, evidence mapping, a visualization method, was utilized to present the trends and gaps in the risk of bias of SRs and the evidence outcomes.

However, this study also has some limitations. Firstly, the language was restricted to English or Chinese. Literature reviews in other languages were not included, causing a potential language bias. Secondly, due to the sudden onset of COVID-19, a large number of original studies showed poor methodological quality, which affected the internal authenticity of the outcomes of SRs. Thirdly, there were some differences in the clinical trial inclusion criteria of each SR: some included retrospective studies instead of real prospective randomized controlled trials. Moreover, we did not include one living SR; therefore, the results of several recent studies were not included in our analysis.

## Conclusion

In conclusion, most SRs of TCM combined with WM approaches for COVID-19 were rated at moderate confidence through AMSTAR 2 and rated at low risk by the ROBIS tool. Most outcomes were graded as moderate quality using the GRADE system. Overall, the results suggested that TCM + WM approaches had advantages in efficacy, laboratory, and clinical symptom outcomes of COVID-19 compared to the WM approach alone. However, to better guide clinical practice in the future, the methodological quality of SRs still needs to be further improved, and the methodological design of the RCTs and OSs included in SRs also needs to be improved.

## Data Availability

The original contributions presented in the study are included in the article/Supplementary Material. Further inquiries can be directed to the corresponding authors.

## Abbreviations

COVID-19, coronavirus disease 2019; CBM, China Biology Medicine; CNKI, China National Knowledge Infrastructure; CIs, confidence intervals; CSFs, colony-stimulating factors; CXCL, C-X-C motif chemokine; GRADE, Grading of Recommendations, Assessment, Development and Evaluation; IL, interleukin; IFN, interferon; MCP, monocyte chemoattractant protein; MIP, macrophage inflammatory protein; MAs, meta-analyses; NMA, network meta-analysis; NCP-D and T program, New Coronavirus Pneumonia Diagnosis and Treatment program; OSs, observational studies; PRIO-harms, Preferred Reporting Items for Overviews of Systematic Reviews including harms; RCTs, randomized controlled trials; ROBIS, Risk of Bias in Systematic Review; SARS-CoV-2, severe acute respiratory syndrome coronavirus 2; SRs, systematic reviews; TNF-α, tumor necrosis factor α; TCM, traditional Chinese medicine; WHO, World Health Organization; WM, Western medicine.
