# Supplementary material for: Evidence Mapping of 23 Systematic Reviews of Traditional Chinese Medicine Combined With Western Medicine Approaches for COVID-19
Source: Front Pharmacol. 2022 Feb 7;12:807491. doi: 10.3389/fphar.2021.807491 (PMC8860227; doi:10.3389/fphar.2021.807491)
Supplement: Supplementary file 1 [file Table1.docx]

**Supplementary Material 1. The search strategy of PubMed database.**

| #1 | "COVID-19" [Mesh] OR "SARS-CoV-2" [Mesh] OR "SARS-CoV-2 variants" [Supplementary Concept] |
| --- | --- |
| #2 | COVID-19 [Title/Abstract] OR COVID 19 [Title/Abstract] OR 2019-nCov [Title/Abstract] OR SARS-CoV-2 [Title/Abstract] OR 2019 novel coronavirus [Title/Abstract] OR coronavirus disease 2019 [Title/Abstract] OR coronavirus disease-19 [Title/Abstract] OR "severe acute respiratory syndrome coronavirus 2" [Title/Abstract] OR "new coronavirus" [Title/Abstract] |
| #3 | #1 OR #2 |
| #4 | (("Medicine, East Asian Traditional" [Mesh]) OR "Medicine, Chinese Traditional" [Mesh]) OR "Plants, Medicinal" [Mesh]) |
| #5 | Traditional Chinese Medicine [Title/Abstract] OR Chinese drug [Title/Abstract] OR herb* [Title/Abstract] OR Chinese medicine [Title/Abstract] |
| #6 | #4 OR #5 |
| #7 | ("Systematic Review" [Publication Type] OR "Systematic Reviews as Topic" [Mesh]) OR ("Meta-Analysis" [Publication Type] OR "Meta-Analysis as Topic" [Mesh]) |
| #8 | systematic review [Title/Abstract] OR systematic reviews [Title/Abstract] OR meta-analysis [Title/Abstract] OR meta-analyses [Title/Abstract] OR meta analysis [Title/Abstract] OR meta analyses [Title/Abstract] OR metaanalysis [Title/Abstract] OR metaanalyses [Title/Abstract] OR data pooling [Title/Abstract] OR data poolings [Title/Abstract] OR overview clinical trial [Title/Abstract] OR Clinical Trial Overview [Title/Abstract] OR Clinical Trial Overviews [Title/Abstract] OR pool analysis [Title/Abstract] OR pool analyses [Title/Abstract] |
| #9 | #7 OR #8 |
| #10 | #3 AND #6 AND #9 |
