# Supplementary material for: Evidence Mapping of 23 Systematic Reviews of Traditional Chinese Medicine Combined With Western Medicine Approaches for COVID-19
Source: Front Pharmacol. 2022 Feb 7;12:807491. doi: 10.3389/fphar.2021.807491 (PMC8860227; doi:10.3389/fphar.2021.807491)
Supplement: Supplementary file 4 [file Table3.docx]

**Supplementary Material 3. The name and composition of each TCM reported in the original studies of the included SRs.**

| **Name of TCM** | **Sources** | **Chinese name** | **Scientific name, family and source** | **Quality control reported? (Y/N)** |
| --- | --- | --- | --- | --- |
| Lianhua Qingwen granules | Beijing Yiling Pharmaceutical Co., Ltd. | Lianqiao | Forsythia suspensa (Thunb.) Vahl [Oleaceae; Forsythiae suspensa fruit] | Y- Prepared according to Pharmacopoeia of the People’s Republic of China; Z20100040. |
|  |  | Jinyinhua | Lonicera japonica Thunb. [Caprifoliaceae; Lonicerae japonicae dry buds or with blooming flowers] |  |
|  |  | Mahuang | Ephedra sinica Stapf [Ephedraceae; Ephedrae sinicae radix] |  |
|  |  | Xingren | Prunus armeniaca L. [Rosaceae; Prunus armeniacae dry mature seeds] |  |
|  |  | Banlangen | Isatis tinctoria subsp. tinctoria [Brassicaceae; Isatis tinctoriae radix et rhizoma] |  |
|  |  | Guanzhong | Dryopteris crassirhizoma Nakai [Polypodiaceae; Dryopteris crassirhizomae radix et rhizoma] |  |
|  |  | Yuxingcao | Houttuynia cordata Thunb. [Saururaceae; Houttuynia cordata fresh whole grass or dry aboveground parts] |  |
|  |  | Huoxiang | Pogostemon cablin (Blanco) Benth. [Lamiaceae; Pogostemon cablin dry aboveground parts] |  |
|  |  | Dahuang | Rheum palmatum L. [Polygonaceae; Rheum palmatum radix et rhizoma] |  |
|  |  | Hongjingtian | Rhodiola chrysanthemifolia subsp. sacra (Raym. - Hamet) H.Ohba [Crassulaceae; Rhodiola chrysanthemifolia radix et rhizoma] |  |
|  |  | Bohe | Mentha canadensis L. [Lamiaceae; Mentha canadensis whole grasses] |  |
|  |  | Shigao | Gypsum Fibrosum [Mineral Drugs] |  |
|  |  | Gancao | Glycyrrhiza uralensis Fisch. ex DC. [Fabaceae; Glycyrrhiza uralensis radix et rhizoma] |  |
| Lianhua Qingwen capsule | Shijiazhuang Yiling Pharmaceutical Co., Ltd. | Lianqiao | Forsythia suspensa (Thunb.) Vahl [Oleaceae; Forsythia suspensa fruit] | Y- Prepared according to Pharmacopoeia of the People’s Republic of China; Z20040063. |
|  |  | Jinyinhua | Lonicera japonica Thunb. [Caprifoliaceae; Lonicera japonica dry buds or with blooming flowers] |  |
|  |  | Mahuang | Ephedra sinica Stapf [Ephedraceae; Ephedra sinica radix] |  |
|  |  | Kuxingren | Prunus armeniaca L. [Rosaceae; Prunus armeniaca dry mature seeds] |  |
|  |  | Shigao | Gypsum Fibrosum [Mineral Drugs] |  |
|  |  | Banlangen | Isatis tinctoria subsp. tinctoria [Brassicaceae; Isatis tinctoria radix et rhizoma] |  |
|  |  | Guanzhong | Dryopteris crassirhizoma Nakai [Polypodiaceae; Dryopteris crassirhizoma radix et rhizoma] |  |
|  |  | Yuxingcao | Houttuynia cordata Thunb. [Saururaceae; Houttuynia cordata fresh whole grass or dry aboveground parts] |  |
|  |  | Huoxiang | Pogostemon cablin (Blanco) Benth. [Lamiaceae; Pogostemon cablin dry aboveground parts] |  |
|  |  | Dahuang | Rheum palmatum L. [Polygonaceae; Rheum palmatum radix et rhizoma] |  |
|  |  | Hongjingtian | Rhodiola chrysanthemifolia subsp. sacra (Raym.-Hamet) H.Ohba [Crassulaceae; Rhodiola chrysanthemifolia radix et rhizoma] |  |
|  |  | Bohe | Mentha canadensis L. [Lamiaceae; Mentha canadensis whole grasses] |  |
|  |  | Gancao | Glycyrrhiza uralensis Fisch. ex DC. [Fabaceae; Glycyrrhiza uralensis radix et rhizoma] |  |
| Shufeng Jiedu capsule | Anhui Jiren Pharmaceutical Co., Ltd. | Huzhang | Reynoutria japonica Houtt. [Polygonaceae; Reynoutria japonica radix et rhizoma] | Y- Prepared according to Pharmacopoeia of the People’s Republic of China; Z20090047. |
|  |  | Lianqiao | Forsythia suspensa (Thunb.) Vahl [Oleaceae; Forsythia suspensa fruit] |  |
|  |  | Banlangen | Isatis tinctoria subsp. tinctoria [Brassicaceae; Isatis tinctoria radix et rhizoma] |  |
|  |  | Chaihu | Bupleurum chinense DC. [Apiaceae; Bupleurum chinense radix et rhizoma] |  |
|  |  | Baijiangcao | Patrinia scabiosifolia Link [Caprifoliaceae; Patrinia scabiosifolia Rooted whole grass] |  |
|  |  | Mabiancao | Verbena officinalis L. [Verbenaceae; Verbena officinalis whole plant] |  |
|  |  | Lugen | Phragmites australis (Cav.) Trin. ex Steud. [Poaceae; Phragmites australis radix et rhizoma] |  |
|  |  | Gancao | Glycyrrhiza uralensis Fisch. ex DC. [Fabaceae; Glycyrrhiza uralensis radix et rhizoma] |  |
| Jinhua Qinggan granules | Juxiechang Beijing Pharmaceutical Co., Ltd. | Jinyinhua | Lonicera japonica Thunb. [Caprifoliaceae; Lonicera japonica dry buds or with blooming flowers] | Y- Prepared according to Pharmacopoeia of the People’s Republic of China; Z20160001. |
|  |  | Shigao | Gypsum Fibrosum [Mineral Drugs] |  |
|  |  | Mahuang | Ephedra sinica Stapf [Ephedraceae; Ephedra sinica radix] |  |
|  |  | Kuxingren | Prunus armeniaca L. [Rosaceae; Prunus armeniaca dry mature seeds] |  |
|  |  | Huangqin | Scutellaria baicalensis Georgi [Lamiaceae; Scutellaria baicalensis radix et rhizoma] |  |
|  |  | Lianqiao | Forsythia suspensa (Thunb.) Vahl [Oleaceae; Forsythia suspensa fruit] |  |
|  |  | Zhebeimu | Fritillaria thunbergii Miq. [Liliaceae; Fritillaria thunbergii radix et rhizoma] |  |
|  |  | Zhimu | Anemarrhena asphodeloides Bunge [Asparagaceae; Anemarrhena asphodeloides radix et rhizoma] |  |
|  |  | Niubangzi | Arctium lappa L. [Asteraceae; Arctium lappa dry ripe fruits] |  |
|  |  | Qinghao | Artemisia annua L. [Asteraceae; Artemisia annua whole grasses] |  |
|  |  | Bohe | Mentha canadensis L. [Lamiaceae; Mentha canadensis whole grasses] |  |
|  |  | Gancao | Glycyrrhiza uralensis Fisch. ex DC. [Fabaceae; Glycyrrhiza uralensis radix et rhizoma] |  |
| Lianhua Qingke granules | Shijiazhuang Yiling Pharmaceutical Co., Ltd. | Mahuang | Ephedra sinica Stapf [Ephedraceae; Ephedra sinica radix] | Batch No. 2020LCKY-003. |
|  |  | Shigao | Gypsum Fibrosum [Mineral Drugs] |  |
|  |  | Lianqiao | Forsythia susp4ensa (Thunb.) Vahl [Oleaceae; Forsythia suspensa fruit] |  |
|  |  | Huangqin | Scutellaria baicalensis Georgi [Lamiaceae; Scutellaria baicalensis radix et rhizoma] |  |
|  |  | Sangbaipi | Morus alba L. [Moraceae; Morus alba Dry root bark] |  |
|  |  | Kuxingren | Prunus armeniaca L. [Rosaceae; Prunus armeniaca dry mature seeds] |  |
|  |  | Qianhu | Angelica decursiva (Miq.) Franch. & Sav. [Apiaceae; Angelica decursiva radix et rhizoma] |  |
|  |  | Banxia | Pinellia cordata N.E.Br. [Araceae; Pinellia cordata radix et rhizoma] |  |
|  |  | Chenpi | Citrus aurantium L. [Rutaceae; Citrus aurantium Dry ripe peel] |  |
|  |  | Zhebeimu | Fritillaria thunbergii Miq. [Liliaceae; Fritillaria thunbergii radix et rhizoma] |  |
|  |  | Niubangzi | Arctium lappa L. [Asteraceae; Arctium lappa dry ripe fruits] |  |
|  |  | Jinyinhua | Lonicera japonica Thunb. [Caprifoliaceae; Lonicera japonica Thunb dry buds or with blooming flowers] |  |
|  |  | Dahuang | Rheum palmatum L. [Polygonaceae; Rheum palmatum radix et rhizoma] |  |
|  |  | Jiegeng | Platycodon grandifloras (Jacq.) A. DC. [Campanulaceae, Platycodon grandifloras radix et rhizoma] |  |
|  |  | Gancao | Glycyrrhiza uralensis Fisch. ex DC. [Fabaceae; Glycyrrhiza uralensis radix et rhizoma] |  |
| Xuebijing Injection | Tianjin Hongri Pharmaceutical Co., Ltd. | Honghua | Carthamus tinctorius L. [Asteraceae; Carthamus tinctorius dried flower] | Y- Prepared according to Pharmacopoeia of the People’s Republic of China; Z20040033. |
|  |  | Chishao | Paeonia lactiflora Pall. [Paeoniaceae; Paeonia iactiflora radix et rhizoma] |  |
|  |  | Chuanxiong | Conioselinum anthriscoides 'Chuanxiong' [Apiaceae; Conioselinum anthriscoides radix et rhizoma] |  |
|  |  | Danshen | Salvia miltiorrhiza Bunge [Lamiaceae; Salvia miltiorrhiza radix et rhizoma] |  |
|  |  | Danggui | Angelica sinensis (Oliv.) Diels [Apiaceae; Angelica sinensis radix et rhizoma] |  |
| Jinyinhua oral | Zhenao Honeysuckle Pharmaceutical Co., Ltd. | Jinyinhua | Lonicera japonica Thunb. [Caprifoliaceae; Lonicera japonica dry buds or with blooming flowers] | Batch No. 19118 |
| Shuanghuanglian oral liquid | Heilongjiang Linbao Pharmaceutical Co., Ltd. | Jinyinhua | Lonicera japonica Thunb. [Caprifoliaceae; Lonicera japonica dry buds or with blooming flowers] | Y- Prepared according to Pharmacopoeia of the People’s Republic of China; Z23020924. |
|  |  | Huangqin | Scutellaria baicalensis Georgi [Lamiaceae; Scutellaria baicalensis radix et rhizoma] |  |
|  |  | Lianqiao | Forsythia suspensa (Thunb.) Vahl [Oleaceae; Forsythia suspensa fruit] |  |
| Huoxiang zhengqi oral liquid | Beijing Yadong Biopharmaceutical Co., Ltd | Cangzhu | Atractylodes lancea (Thunb.) DC. [Asteraceae; Atractylodes lancea radix et rhizoma] | Y- Prepared according to Pharmacopoeia of the People’s Republic of China; Z11020483 |
|  |  | Chenpi | Citrus aurantium L. [Rutaceae; Citrus aurantium Dry ripe peel] |  |
|  |  | Houpo | Magnolia officinalis Rehder & E.H.Wilson [Magnoliacea; Magnolia officinalis root bark] |  |
|  |  | Baizhi | Angelica dahurica (Hoffm.) Benth. & Hook.f. ex Franch. & Sav. [Apiaceae, Angelica dahurica radix et rhizoma] |  |
|  |  | Fuling | Poria cocos (Schw.) Wolf. [Ployporaceae; Poria Dry sclerotium |  |
|  |  | Binlang | Areca catechu L. [Arecaceae; Areca catechu dry mature seeds] |  |
|  |  | Banxia | Pinellia cordata N.E.Br. [Araceae; Pinellia cordata radix et rhizoma] |  |
|  |  | Gancao | Glycyrrhiza uralensis Fisch. ex DC. [Fabaceae; Glycyrrhiza uralensis radix et rhizoma] |  |
|  |  | Huoxiang | Pogostemon cablin (Blanco) Benth. [Lamiaceae; Pogostemon cablin dry aboveground parts] |  |
|  |  | Zisuye | Perilla frutescens (L.) Britton [Lamiaceae; Perilla frutescens whole plants] |  |
| Reyanning Mixture | Tsinghua Deren Xi ' an Happiness Pharmaceutical Co., Ltd. | Pugongying | Taraxacum mongolicum Hand. - Mazz. [Asteraceae, Taraxacum mongolicum whole grasses] | Y- Prepared according to Pharmacopoeia of the People’s Republic of China; Z20050493. |
|  |  | Huzhang | Reynoutria japonica Houtt. [Polygonaceae; Reynoutria japonica radix et rhizoma] |  |
|  |  | Baijiangcao | Patrinia scabiosifolia Link [Caprifoliaceae; Patrinia scabiosifolia Rooted whole grass] |  |
|  |  | Banzhilian | Scutellaria barbata D.Don [Lamiaceae; Scutellaria barbata whole grasses] |  |
| Yupingfeng granules | Sinopharm Guangdong Global Pharmaceutical Co., Ltd. | Huangqi | Astragalus mongholicus Bunge [Fabaceae; Astragalus mongholicus radix et rhizoma] | Y- Prepared according to Pharmacopoeia of the People’s Republic of China; Z10930036. |
|  |  | Fangfeng | Saposhnikovia divaricate (Turcz. ex Ledeb.) Schischk. [Apiaceae; Saposhnikovia divaricate radix et rhizoma] |  |
|  |  | Baizhu | Atractylodes macrocephala Koidz. [Asteraceae; Atractylodes macrocephala radix et rhizoma] |  |
| Toujie Quwen granules | Guangdong Yifang Pharmaceutical Co., Ltd. | Lianqiao | Forsythia suspensa (Thunb.) Vahl [Oleaceae; Forsythia suspensa fruit] | Batch No. 9115313. |
|  |  | Shancigu | Cremastra appendiculata (D.Don) Makino [Orchidaceae; Cremastra appendiculata radix et rhizoma] |  |
|  |  | Jinyinhua | Lonicera japonica Thunb. [Caprifoliaceae; Lonicera japonica dry buds or with blooming flowers] |  |
|  |  | Huangqin | Scutellaria baicalensis Georgi [Lamiaceae; Scutellaria baicalensis radix et rhizoma] |  |
|  |  | Daqingye | Isatis tinctoria subsp. tinctoria [Brassicaceae; Isatis tinctoria subsp Dry leaves] |  |
|  |  | Chaihu | Bupleurum chinense DC. [Apiaceae; Bupleurum chinense radix et rhizoma] |  |
|  |  | Qinghao | Artemisia annua L. [Asteraceae; Artemisia annua whole grasses] |  |
|  |  | Chantui | Cryptotympana pustulata F. [Cicididae; Cryptotympana pustulata shell] |  |
|  |  | Qianhu | Angelica decursiva (Miq.) Franch. & Sav. [Apiaceae; Angelica decursiva radix et rhizoma] |  |
|  |  | Chuanbeimu | Fritillaria cirrhosa D.Don [Liliaceae; Fritillaria cirrhosa radix et rhizoma] |  |
|  |  | Zhebeimu | Fritillaria thunbergii Miq. [Liliaceae; Fritillaria thunbergii cirrhosa radix et rhizoma] |  |
|  |  | Fuling | Poria cocos (Schw.) Wolf. [Ployporaceae; Poria Dry sclerotium |  |
|  |  | Wumei | Prunus mume (Siebold) Siebold & Zucc. [Rosaceae; Prunus mume Dry ripe fruit] |  |
|  |  | Xuanshen | Scrophularia ningpoensis Hemsl. [Scrophulariaceae; Scrophularia ningpoensis radix et rhizoma] |  |
|  |  | Huangqi | Astragalus mongholicus Bunge [Fabaceae; Astragalus mongholicus radix et rhizoma] |  |
|  |  | Taizisen | Pseudostellaria heterophylla (Miq.) Pax [Caryophyllaceae; Pseudostellaria heterophylla radix et rhizoma] |  |
| Qingfei Touxie Fuzheng Recipe | Non-proprietary medicines | Mahuang | Ephedra sinica Stapf [Ephedraceae; Ephedra sinica radix] | Not reported. |
|  |  | Shigao | Gypsum Fibrosum [Mineral Drugs] |  |
|  |  | Xingren | Prunus armeniaca L. [Rosaceae; Prunus armeniaca dry mature seeds] |  |
|  |  | Jinyinhua | Lonicera japonica Thunb. [Caprifoliaceae; Lonicera japonica dry buds or with blooming flowers] |  |
|  |  | Lianqiao | Forsythia suspensa (Thunb.) Vahl [Oleaceae; Forsythia suspensa fruit] |  |
|  |  | Lugen | Phragmites australis (Cav.) Trin. ex Steud. [Poaceae; Phragmites australis radix et rhizoma] |  |
|  |  | Yiyiren | Coix lacryma-jobi L. [Poaceae; Coix lacryma-jobi dry ripe kernels] |  |
|  |  | Jiangcan | Bombyx mori L. [Bombycidae; Bombyx mori dried body] |  |
|  |  | Chantui | Cryptotympana pustulata F. [Cicididae; Cryptotympana pustulata shell] |  |
|  |  | Huzhang | Reynoutria japonica Houtt. [Polygonaceae; Reynoutria japonica radix et rhizoma] |  |
|  |  | Jianghuang | Curcuma longa L. [Zingiberaceae; Curcuma longa radix et rhizoma] |  |
|  |  | Baishao | Paeonia lactiflora Pall. [Paeoniaceae, Paeonia lactiflora radix et rhizoma] |  |
|  |  | Taizisen | Pseudostellaria heterophylla (Miq.) Pax [Caryophyllaceae; Pseudostellaria heterophylla radix et rhizoma] |  |
|  |  | Gancao | Glycyrrhiza uralensis Fisch. ex DC. [Fabaceae; Glycyrrhiza uralensis radix et rhizoma] |  |
| Qingfeipaidu decoction | Non-proprietary medicines | Mahuang | Ephedra sinica Stapf [Ephedraceae; Ephedra sinica radix] | Not reported. |
|  |  | Xingren | Prunus armeniaca L. [Rosaceae; Prunus armeniaca dry mature seeds] |  |
|  |  | Shigao | Gypsum Fibrosum [Mineral Drugs] |  |
|  |  | Guizhi | Neolitsea cassia (L.) Kosterm. [Lauraceae, Neolitsea cassia dry twigs] |  |
|  |  | Ze xie | Alisma plantago-aquatica subsp. orientale (Sam.) Sam. [Alismataceae; Alisma plantago radix et rhizoma] |  |
|  |  | Zhuling | Polyporus umbellatus (Pers.) Fries. [Polyporaceae; Polyporus umbellatus] |  |
|  |  | Baizhu | Atractylodes macrocephala Koidz. [Asteraceae; Atractylodes macrocephala radix et rhizoma] |  |
|  |  | Fuling | Poria cocos (Schw.) Wolf. [Ployporaceae; Poria Dry sclerotium |  |
|  |  | Caihu | Bupleurum chinense DC. [Apiaceae; Bupleurum chinense radix et rhizoma] |  |
|  |  | Huang1in | Scutellaria baicalensis Georgi [Lamiaceae; Scutellaria baicalensis radix et rhizoma] |  |
|  |  | Banxia | Pinellia cordata N.E.Br. [Araceae; Pinellia cordata radix et rhizoma] |  |
|  |  | Shengjiang | Zingiber officinale Roscoe [Zingiberaceae; Zingiber officinale radix et rhizoma] |  |
|  |  | Ziwuan | Aster tataricus L.f. [Asteraceae; Aster tataricus radix et rhizoma] |  |
|  |  | Kuandonghua | Tussilago farfara L. [Asteraceae; Tussilago farfara dried bud] |  |
|  |  | Shegan | Iris domestica (L.) Goldblatt & Mabb. [Iridaceae; Iris domestica radix et rhizoma] |  |
|  |  | Xixin | Asarum heterotropoides F.Schmidt [Aristolochiaceae; Asarum heterotropoides radix et rhizoma] |  |
|  |  | Shanyao | Dioscorea oppositifolia L. [Dioscoreaceae; Dioscorea oppositifolia radix et rhizoma] |  |
|  |  | Zhishi | Citrus aurantium L. [Rutaceae; Citrus aurantium Dry young fruit] |  |
|  |  | Chenpi | Citrus aurantium L. [Rutaceae; Citrus aurantium Dry ripe peel] |  |
|  |  | Huoxiang | Pogostemon cablin (Blanco) Benth. [Lamiaceae; Pogostemon cablin dry aboveground parts] |  |
|  |  | Gancao | Glycyrrhiza uralensis Fisch. ex DC. [Fabaceae; Glycyrrhiza uralensis radix et rhizoma] |  |
| Modified Dayuan decoction | Non-proprietary medicines | Mahuang | Ephedra sinica Stapf [Ephedraceae; Ephedra sinica radix] | Not reported. |
|  |  | Xingren | Prunus armeniaca L. [Rosaceae; Prunus armeniaca dry mature seeds] |  |
|  |  | Shigao | Gypsum Fibrosum [Mineral Drugs] |  |
|  |  | Gualou | Trichosanthes kirilowii Maxim. [Cucurbitaceae; Trichosanthes kirilowii Dry ripe fruit] |  |
|  |  | Tinglizi | Lepidium apetalum Willd. [Brassicaceae; Lepidium apetalum dry mature seeds] |  |
|  |  | Taoren | Prunus persica (L.) Batsch [Rosaceae; Prunus persica dry mature seeds] |  |
|  |  | Caoguo | Lanxangia tsao-ko (Crevost & Lemarié) M.F.Newman & Skornick. [Zingiberaceae; Lanxangia tsao-ko Dry ripe fruit] |  |
|  |  | Binlang | Areca catechu L. [Arecaceae; Areca catechu dry mature seeds] |  |
|  |  | Cangzhu | Atractylodes lancea (Thunb.) DC. [Asteraceae; Atractylodes lancea radix et rhizoma] |  |
| Maxing Xuanfei Jiedu Decoction | Non-proprietary medicines Formula | Mahuang | Ephedra sinica Stapf [Ephedraceae; Ephedra sinica radix] | Not reported. |
|  |  | Xingren | Prunus armeniaca L. [Rosaceae; Prunus armeniaca dry mature seeds] |  |
|  |  | Shigao | Gypsum Fibrosum [Mineral Drugs] |  |
|  |  | Zhebeimu | Fritillaria thunbergii Miq. [Liliaceae; Fritillaria thunbergii cirrhosa radix et rhizoma] |  |
|  |  | Jiangcan | Bombyx mori L. [Bombycidae; Bombyx mori dried body] |  |
|  |  | Chantui | Cryptotympana pustulata F. [Cicididae; Cryptotympana pustulata shell] |  |
|  |  | Jianghuang | Curcuma longa L. [Zingiberaceae; Curcuma longa radix et rhizoma] |  |
|  |  | Jiegeng | Platycodon grandifloras (Jacq.) A. DC. [Campanulaceae; Platycodon grandifloras radix et rhizoma] |  |
|  |  | Zhiqiao | Citrus aurantium L. [Rutaceae; Citrus aurantium Dry immature fruits] |  |
|  |  | Caoguo | Lanxangia tsao-ko (Crevost & Lemarié) M.F.Newman & Skornick. [Zingiberaceae; Lanxangia tsao-ko Dry ripe fruit] |  |
|  |  | Doukou | Wurfbainia vera (Blackw.) Skornick. & A.D.Poulsen [Zingiberaceae; Wurfbainia vera Dry ripe fruit] |  |
| Maxingshigan-dayuanyin  decoction | Non-proprietary medicines | Mahuang | Ephedra sinica Stapf [Ephedraceae; Ephedra sinica radix] | Not reported. |
|  |  | Xingren | Prunus armeniaca L. [Rosaceae; Prunus armeniaca dry mature seeds] |  |
|  |  | Shigao | Gypsum Fibrosum [Mineral Drugs] |  |
|  |  | Gualou | Trichosanthes kirilowii Maxim. [Cucurbitaceae; Trichosanthes kirilowii Dry ripe fruit] |  |
|  |  | Qianhu | Angelica decursiva (Miq.) Franch. & Sav. [Apiaceae; Angelica decursiva radix et rhizoma] |  |
|  |  | Chuanbeimu | Fritillaria cirrhosa D.Don [Liliaceae; Fritillaria cirrhosa radix et rhizoma] |  |
|  |  | Zhuye | Phyllostachys nigra (Lodd. ex Lindl.) Munro [Poaceae; Phyllostachys nigra leaves] |  |
|  |  | Binlang | Areca catechu L. [Arecaceae; Areca catechu dry mature seeds] |  |
|  |  | Houpo | Magnolia officinalis Rehder & E.H.Wilson [Magnoliacea; Magnolia officinalis root bark] |  |
|  |  | Zhimu | Anemarrhena asphodeloides Bunge [Asparagaceae; Anemarrhena asphodeloides radix et rhizoma] |  |
|  |  | Chishao | Paeonia lactiflora Pall. [Paeoniaceae; Paeonia iactiflora radix et rhizoma] |  |
|  |  | Huangqin | Scutellaria baicalensis Georgi [Lamiaceae; Scutellaria baicalensis radix et rhizoma] |  |
|  |  | Caoguo | Lanxangia tsao-ko (Crevost & Lemarié) M.F.Newman & Skornick. [Zingiberaceae; Lanxangia tsao-ko Dry ripe fruit] |  |
|  |  | Gancao | Glycyrrhiza uralensis Fisch. ex DC. [Fabaceae; Glycyrrhiza uralensis radix et rhizoma] |  |
|  |  | Fuzi | Aconitum carmichaeli Debeaux [Ranunculaceae, Aconitum carmichaeli radix et rhizoma] |  |
|  |  | Qingdai | Isatis tinctoria subsp. tinctoria [Brassicaceae; Isatis tinctoria Dry powder, lump or particle] |  |
| Shiduyufei decoction | Non-proprietary medicines | Xingren | Prunus armeniaca L. [Rosaceae; Prunus armeniaca dry mature seeds] | Not reported. |
|  |  | Huashi | Talcum [Mineral Drugs] |  |
|  |  | Cangzhu | Atractylodes lancea (Thunb.) DC. [Asteraceae; Atractylodes lancea radix et rhizoma] |  |
|  |  | Baizhi | Atractylodes macrocephala Koidz. [Asteraceae; Atractylodes macrocephala radix et rhizoma] |  |
|  |  | Banxia | Pinellia cordata N.E.Br. [Araceae; Pinellia cordata radix et rhizoma] |  |
|  |  | Huoxiang | Pogostemon cablin (Blanco) Benth. [Lamiaceae; Pogostemon cablin dry aboveground parts] |  |
|  |  | Fuling | Poria cocos (Schw.) Wolf. [Ployporaceae; Poria Dry sclerotium |  |
|  |  | Mahuang | Ephedra sinica Stapf [Ephedraceae; Ephedra sinica radix] |  |
|  |  | Dahuang | Rheum palmatum L. [Polygonaceae; Rheum palmatum radix et rhizoma] |  |
|  |  | Chantui | Cryptotympana pustulata F. [Cicididae; Cryptotympana pustulata shell] |  |
|  |  | Niubangzi | Arctium lappa L. [Asteraceae; Arctium lappa dry ripe fruits] |  |
|  |  | Gancao | Glycyrrhiza uralensis Fisch. ex DC. [Fabaceae; Glycyrrhiza uralensis radix et rhizoma] |  |
| Pneumonia  No.1 Prescription | Non-proprietary medicines | Qinghao | Artemisia annua L. [Asteraceae; Artemisia annua whole grasses] | Not reported. |
|  |  | Huangqi | Astragalus mongholicus Bunge [Fabaceae; Astragalus mongholicus radix et rhizoma] |  |
|  |  | Shancigu | Cremastra appendiculata (D.Don) Makino [Orchidaceae; Cremastra appendiculata radix et rhizoma] |  |
|  |  | Lianqiao | Forsythia suspensa (Thunb.) Vahl [Oleaceae; Forsythia suspensa fruit] |  |
|  |  | Huangqin | Scutellaria baicalensis Georgi [Lamiaceae; Scutellaria baicalensis radix et rhizoma] |  |
|  |  | Jinyinhua | Lonicera japonica Thunb. [Caprifoliaceae; Lonicera japonica dry buds or with blooming flowers] |  |
|  |  | Daqingye | Isatis tinctoria subsp. tinctoria [Brassicaceae; Isatis tinctoria subsp Dry leaves] |  |
|  |  | Chaihu | Bupleurum chinense DC. [Apiaceae; Bupleurum chinense radix et rhizoma] |  |
|  |  | Chantui | Cryptotympana pustulata F. [Cicididae; Cryptotympana pustulata shell] |  |
|  |  | Qianhu | Angelica decursiva (Miq.) Franch. & Sav. [Apiaceae; Angelica decursiva radix et rhizoma] |  |
|  |  | Chuanbeimu | Fritillaria cirrhosa D.Don [Liliaceae; Fritillaria cirrhosa radix et rhizoma] |  |
|  |  | Zhebeimu | Fritillaria thunbergii Miq. [Liliaceae; Fritillaria thunbergii cirrhosa radix et rhizoma] |  |
|  |  | Wumei | Prunus mume (Siebold) Siebold & Zucc. [Rosaceae; Prunus mume Dry ripe fruit] |  |
|  |  | Xuanshen | Scrophularia ningpoensis Hemsl. [Scrophulariaceae; Scrophularia ningpoensis radix et rhizoma] |  |
|  |  | Fuling | Poria cocos (Schw.) Wolf. [Ployporaceae; Poria Dry sclerotium |  |
|  |  | Taizishen | Pseudostellaria heterophylla (Miq.) Pax [Caryophyllaceae; Pseudostellaria heterophylla radix et rhizoma] |  |
