# Supplementary material for: Evidence Mapping of 23 Systematic Reviews of Traditional Chinese Medicine Combined With Western Medicine Approaches for COVID-19
Source: Front Pharmacol. 2022 Feb 7;12:807491. doi: 10.3389/fphar.2021.807491 (PMC8860227; doi:10.3389/fphar.2021.807491)
Supplement: Supplementary file 6 [file Table6.docx]

**Supplementary Material 6. Summary the evidence quality of outcomes of included SRs.**

| **Study ID** | **Outcomes** | **Subgroup** | **Interventions (T/C)** | | **Studies/**  **Sample** | Effect and 95%CI | **Quality of Evidence** |
| --- | --- | --- | --- | --- | --- | --- | --- |
| Xiong, et al., 2020 | CT recovery rate | RCT | (Lianhua Qingwen granules, Qingfei Touxie Fuzheng Recipe, Toujie Quwen granules, Lianhua Qingwen capsule, Chinese Herbal Medicine, Shufeng Jiedu capsule, Reyanning Mixture) + WM | WM | 13 (1402) | OR=1.23, 95%CI (1.15, 1.32) | ⨁⨁⨁◯ MODERATE^*^ |
|  | Mortality | RCT | (Reyanning Mixture, Lianhua Qingwen granules) + WM | WM | 4 (463) | OR=0.34, 95%CI (0.05, 2.18) | ⨁⨁⨁◯ MODERATE^*^ |
|  | Clinical cure rate | RCT | (Lianhua Qingwen granules, Toujie Quwen granules, Lianhua Qingwen capsule, Shufeng Jiedu capsule) + WM | WM | 7 (1523) | OR=1.18, 95%CI (1.13, 1.24) | ⨁⨁⨁◯ MODERATE^*^ |
|  | The rate of ranging from critical to mild cases | RCT | Qingfei Touxie Fuzheng Recipe + WM | WM | 2 (167) | OR=1.34, 95%CI (0.47, 3.80) | ⨁⨁⨁◯ MODERATE^*^ |
|  | The rate of ranging from mild to critical cases | RCT | (Lianhua Qingwen granules, Jinhua Qinggan granules, Lianhua Qingwen capsule, Lianhua Qingke granules, Reyanning Mixture) + WM | WM | 11 (1246) | OR=0.40, 95%CI (0.29, 0.56) | ⨁⨁⨁◯ MODERATE^*^ |
|  | Length of hospital stay | RCT | Chinese Herbal Medicine + WM | WM | 2 (119) | WMD=-1.99, 95%CI (-3.28, -0.70) | ⨁⨁⨁◯ MODERATE^*^ |
|  | The rate of fever reduction | RCT | (Qingfei Touxie Fuzheng Recipe, Jinhua Qinggan granules, Lianhua Qingke granules, Lianhua Qingwen granules) + WM | WM | 5 (388) | RR=1.28, 95%CI (0.98, 1.67) | ⨁⨁◯◯ LOW^*$^ |
|  | Fever reduction time | RCT | (Lianhua Qingwen granules, Lianhua Qingwen capsule, Shufeng Jiedu capsule, Reyanning Mixture) + WM | WM | 10 (1017) | WMD=-1.36, 95% CI (-1.80, -0.93) | ⨁⨁◯◯ LOW^*$^ |
|  | The symptom score of fever | RCT | (Toujie Quwen granules, Chinese Herbal Medicine, Lianhua Qingwen granules) + WM | WM | 3 (885) | WMD=-0.60, 95% CI (-0.69, -0.50) | ⨁⨁◯◯ LOW^*$^ |
|  | The rate of cough reduction | RCT | (Lianhua Qingwen granules, Qingfei Touxie Fuzheng Recipe, Jinhua Qinggan granules) + WM | WM | 6 (322) | RR=1.50, 95%CI (1.26, 1.78) | ⨁⨁⨁◯ MODERATE^*^ |
|  | The symptom score of coughs | RCT | (Toujie Quwen granules, Reyanning Mixture, Lianhua Qingwen granules) + WM | WM | 4 (934) | WMD=-0.78, 95% CI (-1.32, -0.24) | ⨁⨁◯◯ LOW^*$^ |
|  | The disappearing time of cough | RCT | (Lianhua Qingwen granules, Lianhua Qingwen capsule, Shufeng Jiedu capsule) + WM | WM | 6 (698) | WMD=-1.42, 95% CI (-2.82, -0.01) | ⨁⨁◯◯ LOW^*$^ |
|  | The rate of fatigue reduction | RCT | (Lianhua Qingwen granules, Jinhua Qinggan granules) + WM | WM | 5 (307) | RR=1.73, 95%CI (1.39, 2.16) | ⨁⨁⨁◯ MODERATE^*^ |
|  | The symptom score of fatigue | RCT | (Toujie Quwen granules, Chinese Herbal Medcine, Reyanning Mixture, Lianhua Qingwen granules) + WM | WM | 4 (934) | WMD=-0.70, 95% CI (-0.98, -0.42) | ⨁⨁◯◯ LOW^*$^ |
|  | The disappearing time of fatigue | RCT | (Lianhua Qingwen granules, Lianhua Qingwen capsule, Shufeng Jiedu capsule) + WM | WM | 4 (585) | WMD=-1.13, 95% CI (-2.22, -0.04) | ⨁⨁◯◯ LOW^*$^ |
|  | Viral nucleic acid negative conversion rate | RCT | (Lianhua Qingwen granules, Shufeng Jiedu capsule, Reyanning Mixture) + WM | WM | 4 (469) | RR=1.18, 95%CI (1.04, 1.34) | ⨁⨁◯◯ LOW^*$^ |
|  | White blood cell count | RCT | (Toujie Quwen granules, Shufeng Jiedu capsule, Lianhua Qingwen granules) + WM | WM | 5 (1151) | WMD=0.27, 95% CI (-0.22, 0.76) | ⨁⨁◯◯ LOW^*$^ |
|  | Lymphocyte count | RCT | (Toujie Quwen granules, Reyanning Mixture, Lianhua Qingwen granules) + WM | WM | 4 (483) | WMD=0.24, 95% CI (-0.04, 0.51) | ⨁⨁◯◯ LOW^*$^ |
|  | The amount of C-reactive protein | RCT | (Qingfei Touxie Fuzheng Recipe, Toujie Quwen granules, Reyanning Mixture, Lianhua Qingwen granules) + WM | WM | 6 (1100) | WMD=-8.91, 95% CI (-12.56, -5.27) | ⨁⨁◯◯ LOW^*$^ |
|  | Adverse effects | RCT | (Qingfei Touxie Fuzheng Recipe, Jinhua Qinggan granules, Toujie Quwen granules, Lianhua Qingwen granules, Shufeng Jiedu capsule) + WM | WM | 9 (1069) | RR=0.93, 95%CI (0.49, 1.75) | ⨁⨁⨁◯ MODERATE^*^ |
| Liu, et al., 2021 | Overall effective rate | RCT | (Lianhua Qingwen capsule, Lianhua Qingwen granules) + WM | WM | 2 (578) | RR=1.16, 95%CI (1.04, 1.30) | ⨁⨁⨁◯ MODERATE^*^ |
|  | CT recovery rate | RCT | (Lianhua Qingwen capsule, Lianhua Qingwen granules) + WM | WM | 2 (579) | RR=1.21, 95%CI (1.02, 1.43) | ⨁⨁◯◯ LOW^*$^ |
|  | Aggravation rate | RCT | (Lianhua Qingwen capsule, Lianhua Qingwen granules) + WM | WM | 2 (579) | RR=0.59, 95%CI (0.37, 0.94) | ⨁⨁⨁◯ MODERATE^*^ |
| Lin, et al., 2020 | Total effective rate | RCT | (Toujie Quwen granules, Shufeng Jiedu capsule, Lianhua Qingwen granules) + WM | WM | 4 (633) | RR=1.23, 95%CI (1.13, 1.34) | ⨁⨁⨁◯ MODERATE^*^ |
|  | Cough disappearance rate | RCT | (Jinhua Qinggan granules, Lianhua Qingke granules) + WM | WM | 2 (147) | RR=1.45, 95%CI (1.12, 1.89) | ⨁⨁⨁◯ MODERATE^*^ |
|  | Sputum disappearance rate | RCT | (Jinhua Qinggan granules, Lianhua Qingke granules) + WM | WM | 2 (80) | RR=1.73, 95%CI (1.19, 2.50) | ⨁⨁◯◯ LOW^*&^ |
|  | TCM syndrome score of coughs | RCT | (Toujie Quwen granules, Lianhua Qingwen granules) + WM | WM | 3 (433) | MD=-1.18, 95%CI (-1.34, -1.03) | ⨁⨁◯◯ LOW^*$^ |
|  | TCM syndrome score of fever | RCT | (Toujie Quwen granules, Lianhua Qingwen granules) + WM | WM | 3 (433) | MD=-0.62, 95%CI (-0.79, -0.45) | ⨁⨁◯◯ LOW^*$^ |
|  | TCM syndrome score of dry and sore throat | RCT | (Toujie Quwen granules, Lianhua Qingwen granules) + WM | WM | 3 (433) | MD=-0.83, 95%CI (-1.45, -0.20) | ⨁⨁◯◯ LOW^*$^ |
|  | TCM syndrome score of fatigue | RCT | (Toujie Quwen granules, Lianhua Qingwen granules) + WM | WM | 3 (433) | MD=-0.60, 95%CI (-1.04, -0.17) | ⨁⨁◯◯ LOW^*$^ |
|  | White blood cell counts | RCT | (Toujie Quwen granules, Shufeng Jiedu capsule, Lianhua Qingwen granules) + WM | WM | 3 (540) | MD=0.49, 95%CI (0.27, 0.70) | ⨁⨁◯◯ LOW^*$^ |
|  | Lymphocyte counts | RCT | (Toujie Quwen granules, Lianhua Qingwen granules) + WM | WM | 3 (433) | MD=0.27, 95%CI (0.06, 0.47) | ⨁⨁◯◯ LOW^*$^ |
|  | Lymphocyte percentage | RCT | (Toujie Quwen granules, Shufeng Jiedu capsule) + WM | WM | 3 (256) | MD=3.83, 95%CI (1.13, 6.53) | ⨁⨁◯◯ LOW^*$^ |
|  | Total procalcitonin level | RCT | (Toujie Quwen granules, Lianhua Qingwen granules) + WM | WM | 2 (360) | MD=-0.02, 95%CI (-0.02, -0.01) | ⨁⨁⨁◯ MODERATE^*^ |
|  | The level of C-reactive protein | RCT | (Toujie Quwen granules, Lianhua Qingwen granules) + WM | WM | 3 (433) | MD=-6.32, 95%CI (-11.40, -1.23) | ⨁⨁◯◯ LOW^*$^ |
|  | The neutrophil percentage | RCT | Toujie Quwen granules + WM | WM | 1 (65) | MD=-4.52, 95%CI (-5.81, -3.35) | ⨁⨁⨁◯ MODERATE^*^ |
|  | D-Dimer level | RCT | Toujie Quwen granules + WM | WM | 1 (65) | MD=-42.50, 95%CI (-84.55, -0.45) | ⨁⨁⨁◯ MODERATE^*^ |
|  | The rate of relieving anxiety | RCT | Jinhua Qinggan granules + WM | WM | 1 (123) | RR=0.34, 95%CI (-1.14, 1.82) | ⨁⨁⨁◯ MODERATE^*^ |
|  | Fever disappearance time | RCT | Jinhua Qinggan granules+WM | WM | 1 (123) | RR=1.51, 95%CI (1.07, 2.14) | ⨁⨁⨁◯ MODERATE^$^ |
|  | The improvement rate of chest CT | RCT | Lianhua Qingke granules + WM | WM | 1 (57) | RR=1.35, 95%CI (1.05, 1.73) | ⨁⨁⨁◯ MODERATE^*^ |
|  | The oxygenation index | RCT | Lianhua Qingke granules + WM | WM | 1 (57) | MD=73.73, 95%CI (52.75, 94.71) | ⨁⨁⨁◯ MODERATE^*^ |
|  | The absorption rate of lesions based on chest  CT | RCT | Shufeng Jiedu capsule + WM | WM | 1 (200) | RR=1.21, 95%CI (1.05, 1.40) | ⨁⨁⨁◯ MODERATE^*^ |
|  | Hospital discharge rate | RCT | Toujie Quwen granules + WM | WM | 1 (73) | RR=1.42, 95%CI (0.76, 2.62) | ⨁⨁⨁◯ MODERATE^*^ |
|  | Adverse events | RCT | (Jinhua Qinggan granules, Toujie Quwen granules, Shufeng Jiedu capsule, Lianhua Qingwen granules) + WM | WM | 5 (756) | RD=0.06, 95%CI (-0.04, 0.15) | ⨁⨁◯◯ LOW^*$^ |
| Zhou, et al., 2021 | Cure rate | RCT | (Qingfei Touxie Fuzheng Recipe, Touxie Quwen granules, Jinyinhua oral liquid, Lianhua Qingwen capsule) + WM | WM | 6 (976) | RR=1.15, 95%CI (1.04, 1.26) | ⨁⨁◯◯ LOW^*$^ |
|  | The rate of lowering body temperature | RCT | (Lianhua Qingwen granules, Qingfei Touxie Fuzheng Recipe, Lianhua Qingke granules, Pneumonia No.1 Prescription, Jinyinhua oral liquid) + WM | WM | 6 (419) | RR=1.10, 95%CI (0.94, 1.29) | ⨁⨁◯◯ LOW^*$^ |
|  | The rate of relieving cough | RCT | (Lianhua Qingwen granules, Qingfei Touxie Fuzheng Recipe, Lianhua Qingke granules, Pneumonia No.1 Prescription, Jinyinhua oral liquid) + WM | WM | 6 (454) | RR=1.32, 95%CI (1.15, 1.52) | ⨁⨁⨁◯ MODERATE^*^ |
|  | The rate of improvement in chest CT images | RCT | (Qingfei Touxie Fuzheng Recipe, Lianhua Qingwen capsule) + WM | WM | 4 (749) | RR=1.23, 95%CI (1.11, 1.37) | ⨁⨁⨁◯ MODERATE^*^ |
|  | The rate of deterioration of condition | RCT | (Jinhua Qinggan granules, Toujie Quwen granules, Lianhua Qingwen granules, Jinyinhua oral liquid, Lianhua Qingwen capsule) + WM | WM | 6 (751) | RR=0.58, 95%CI (0.43, 0.77) | ⨁⨁⨁◯ MODERATE^*^ |
|  | Adverse effects | RCT | (Jinhua Qinggan granules, Qingfei Touxie Fuzheng Recipe, Jinyinhua oral liquid, Lianhua Qingwen capsule) + WM | WM | 6 (780) | RR=0.81, 95%CI (0.42, 1.57) | ⨁⨁◯◯ LOW^*$^ |
| Shi, et al., 2020 | The remission rate of nausea | RCT + OS | Chinese herbal medicine + WM | WM | 6 (57) | RR=1.04, 95%CI (0.83, 1.30) | ⨁⨁◯◯ LOW^*&^ |
|  |  | RCT | Chinese herbal medicine + WM | WM | 1 (11) | RR=1.11, 95%CI (0.45, 2.77) | ⨁⨁◯◯ LOW^*&^ |
|  |  | OS | Chinese herbal medicine + WM | WM | 5 (46) | RR=1.04, 95%CI (0.82, 1.31) | ⨁⨁◯◯ LOW |
|  | The remission rate of vomiting | RCT + OS | Chinese herbal medicine + WM | WM | 4 (25) | RR=1.00, 95%CI (0.77, 1.29) | ⨁⨁◯◯ LOW^*&^ |
|  |  | RCT | Chinese herbal medicine + WM | WM | 1 (6) | RR=1.00, 95%CI (0.32, 3.10) | ⨁⨁◯◯ LOW^*&^ |
|  |  | OS | Chinese herbal medicine + WM | WM | 3 (19) | RR=1.00, 95%CI (0.77, 1.30) | ⨁⨁◯◯ LOW |
|  | The remission rate of anorexia | RCT + OS | Chinese herbal medicine + WM | WM | 6 (160) | RR=2.09, 95%CI (1.04, 4.19) | ⨁⨁⨁◯ MODERATE^#^ |
|  |  | RCT | Chinese herbal medicine + WM | WM | 3 (70) | RR=1.28, 95%CI (0.77, 2.12) | ⨁⨁◯◯ LOW^*$^ |
|  |  | OS | Chinese herbal medicine + WM | WM | 3 (90) | RR=3.32, 95%CI (1.10, 9.98) | ⨁⨁⨁◯ MODERATE^#^ |
|  | The remission rate of diarrhea | RCT + OS | Chinese herbal medicine + WM | WM | 12 (187) | RR=1.04, 95%CI (0.92, 1.16) | ⨁⨁⨁◯ MODERATE^*^ |
|  |  | RCT | Chinese herbal medicine + WM | WM | 6 (137) | RR=1.09, 95%CI (0.85, 1.39) | ⨁⨁⨁◯ MODERATE^*^ |
|  |  | OS | Chinese herbal medicine + WM | WM | 6 (50) | RR=1.10, 95%CI (0.85, 1.18) | ⨁⨁◯◯ LOW |
|  | The effective rate of ALT returning to normal | RCT + OS | Chinese herbal medicine + WM | WM | 4 (192) | RR=1.23, 95%CI (0.91, 1.65) | ⨁⨁⨁⨁ HIGH |
|  |  | RCT | Chinese herbal medicine + WM | WM | 1 (82) | RR=1.28, 95%CI (1.02, 1.59) | ⨁⨁⨁⨁ HIGH |
|  |  | OS | Chinese herbal medicine + WM | WM | 3 (110) | RR=1.15, 95%CI (0.68, 1.93) | ⨁⨁◯◯ LOW |
|  | The effective rate of AST returning to normal | RCT + OS | Chinese herbal medicine + WM | WM | 4 (196) | RR=1.22, 95%CI (0.92, 1.61) | ⨁⨁⨁⨁ HIGH |
|  |  | RCT | Chinese herbal medicine + WM | WM | 1 (82) | RR=1.21, 95%CI (1.03, 1.42) | ⨁⨁⨁⨁ HIGH |
|  |  | OS | Chinese herbal medicine + WM | WM | 3 (114) | RR=1.25, 95%CI (0.75, 2.10) | ⨁⨁◯◯ LOW |
|  | Aggravation rate of COVID-19 | RCT + OS | Chinese herbal medicine + WM | WM | 28 (3415) | RR=0.43, 95%CI (0.34, 0.55) | ⨁⨁⨁◯ MODERATE^*^ |
|  |  | RCT | Chinese herbal medicine + WM | WM | 12 (1375) | RR=0.46, 95%CI (0.33, 0.64) | ⨁⨁⨁◯ MODERATE^*^ |
|  |  | OS | Chinese herbal medicine + WM | WM | 16 (2040) | RR=0.40, 95%CI (0.28, 0.57) | ⨁⨁◯◯ LOW |
|  | Time to viral assay conversion | RCT + OS | Chinese herbal medicine + WM | WM | 19 (1772) | MD=-3.48, 95%CI (-4.33, -2.64) | ⨁⨁◯◯ LOW^*$^ |
|  |  | RCT | Chinese herbal medicine + WM | WM | 6 (423) | MD=-3.75, 95%CI (-6.19, -1.30) | ⨁⨁◯◯ LOW^*$^ |
|  |  | OS | Chinese herbal medicine + WM | WM | 13 (1349) | MD=-3.44, 95%CI (-4.28, -2.60) | ⨁⨁◯◯ LOW |
| Zeng, et al., 2020 | Disappearance rate of fever | RCT | Lianhua Qingwen + WM | WM | 2 (144) | OR=3.64, 95%CI (1.57, 8.47) | ⨁⨁⨁◯ MODERATE^*^ |
|  | Disappearance rate of cough | RCT | Lianhua Qingwen + WM | WM | 1 (102) | OR=4.22, 95%CI (1.73, 10.26) | ⨁⨁⨁◯ MODERATE^*^ |
|  | Disappearance rate of fatigue | RCT | Lianhua Qingwen + WM | WM | 2 (144) | OR=2.53, 95%CI (1.13, 5.68) | ⨁⨁⨁◯ MODERATE^*^ |
|  | Disappearance rate of muscle pain | RCT | Lianhua Qingwen + WM | WM | 2 (144) | OR=6.97, 95%CI (1.47, 33.01) | ⨁⨁⨁◯ MODERATE^*^ |
|  | Disappearance rate of sputum | RCT | Lianhua Qingwen + WM | WM | 2 (144) | OR=8.82, 95%CI (2.48, 31.41) | ⨁⨁⨁◯ MODERATE^*^ |
|  | Disappearance rate of shortness of breath | RCT | Lianhua Qingwen + WM | WM | 2 (144) | OR=13.08, 95%CI (2.60, 65.91) | ⨁⨁⨁◯ MODERATE^*^ |
|  | Disappearance rate of chest tightness | RCT | Lianhua Qingwen + WM | WM | 2 (144) | OR=7.17, 95%CI (1.83, 28.12) | ⨁⨁⨁◯ MODERATE^*^ |
|  | Disappearance rate of difficulty breathing | RCT | Lianhua Qingwen + WM | WM | 2 (144) | OR=2.82, 95%CI (0.27, 29.18) | ⨁⨁⨁◯ MODERATE^*^ |
|  | Disappearance rate of nausea | RCT | Lianhua Qingwen + WM | WM | 2 (144) | OR=1.21, 95%CI (0.19, 7.81) | ⨁⨁⨁◯ MODERATE^*^ |
|  | Disappearance rate of loss of appetite | RCT | Lianhua Qingwen + WM | WM | 2 (144) | OR=18.07, 95%CI (0.33, 997.88) | ⨁⨁◯◯ LOW^*&^ |
| Liu, et al., 2020 | Clinical Efficacy | RCT + OS | (Shufeng Jiedu capsule, Lianhua Qingwen granules, Toujie Quwen granules) + WM | WM | 4 (479) | RR=1.230, 95%CI (1.113, 1.359) | ⨁⨁⨁◯ MODERATE^*^ |
|  | Cure rate | RCT + OS | (Shufeng Jiedu capsule, Toujie Quwen granules) + WM | WM | 4 (299) | RR=1.604, 95%CI (1.181, 2.177) | ⨁⨁⨁◯ MODERATE^*^ |
|  | Severity illness rate | RCT + OS | Jinhua Qinggan granules + WM | WM | 2 (175) | RR=0.350, 95%CI (0.154, 0.792) | ⨁⨁⨁◯ MODERATE^*^ |
|  | Hospital stays | OS | (Integrated Traditional Chinese and Western Medicine, Chinese Herbal Medicine) + WM | WM | 2 (119) | WMD=-1.99, 95%CI (-3.28, -0.70) | ⨁⨁◯◯ LOW |
|  | Fever disappearance rate | OS | Integrated Traditional Chinese and Western Medicine | WM | 4 (324) | RR=1.320, 95%CI (1.048, 1.663) | ⨁⨁◯◯ LOW |
|  | Cough disappearance rate | OS | Integrated Traditional Chinese and Western Medicine | WM | 4 (283) | RR=1.590, 95%CI (1.122, 2.253) | ⨁⨁◯◯ LOW |
|  | Expectoration disappearance rate | OS | Integrated Traditional Chinese and Western Medicine | WM | 3 (126) | RR=2.549, 95%CI (1.390, 4.678) | ⨁⨁⨁◯ MODERATE^#^ |
|  | Fatigue disappearance rate | OS | Integrated Traditional Chinese and Western Medicine | WM | 3 (175) | RR=1.532, 95%CI (1.137, 2.065) | ⨁⨁◯◯ LOW |
|  | Myalgia disappearance rate | OS | Integrated Traditional Chinese and Western Medicine | WM | 3 (63) | RR=1.783, 95%CI (0.812, 3.913) | ⨁⨁◯◯ LOW |
|  | Chest tightness disappearance rate | OS | Integrated Traditional Chinese and Western Medicine | WM | 3 (81) | RR=2.587, 95%CI (1.506, 4.444) | ⨁⨁⨁◯ MODERATE^#^ |
|  | Nausea disappearance rate | OS | Integrated Traditional Chinese and Western Medicine | WM | 3 (38) | RR=1.132, 95%CI (0.717, 1.787) | ⨁⨁◯◯ LOW |
|  | Anorexia disappearance rate | OS | Integrated Traditional Chinese and Western Medicine | WM | 2 (57) | RR=5.043, 95%CI (1.116, 22.783) | ⨁⨁⨁◯ MODERATE^#^ |
|  | Diarrhea disappearance rate | OS | Integrated Traditional Chinese and Western Medicine | WM | 3 (38) | RR=0.681, 95%CI (0.199, 2.332) | ⨁⨁◯◯ LOW |
|  | Fever disappearance time | OS | Integrated Traditional Chinese and Western Medicine | WM | 5 (425) | WMD=-1.319, 95%CI (-1.842, -0.796) | ⨁⨁◯◯ LOW |
|  | Cough disappearance time | OS | Integrated Traditional Chinese and Western Medicine | WM | 3 (307) | WMD=-0.993, 95%CI (-2.397, -0.532) | ⨁⨁◯◯ LOW |
|  | Fatigue disappearance time | OS | Integrated Traditional Chinese and Western Medicine | WM | 3 (301) | WMD=-1.129, 95%CI (-2.221, -0.037) | ⨁⨁◯◯ LOW |
|  | Nasal congestion disappearance time | OS | Integrated Traditional Chinese and Western Medicine | WM | 2 (270) | WMD=0.033, 95%CI (-0.218, 0.348) | ⨁⨁◯◯ LOW |
|  | Runny nose disappearance time | OS | Integrated Traditional Chinese and Western Medicine | WM | 2 (270) | WMD=-0.800, 95%CI (-2.515, 0.915) | ⨁⨁◯◯ LOW |
|  | The amount of C-reactive protein | OS | Integrated Traditional Chinese and Western Medicine | WM | 4 (326) | WMD=-1.16, 95%CI (-6.96, 4.65) | ⨁⨁◯◯ LOW |
|  | TNF-α level | OS | Integrated Traditional Chinese and Western Medicine | WM | 2 (204) | WMD=-3.13, 95%CI (-4.23, -2.04) | ⨁⨁◯◯ LOW |
|  | Lymphocyte count | OS | Integrated Traditional Chinese and Western Medicine | WM | 2 (237) | WMD=1.59, 95%CI (0.61, 2.58) | ⨁⨁◯◯ LOW |
|  | WBC count | OS | Integrated Traditional Chinese and Western Medicine | WM | 2 (237) | WMD=0.66, 95%CI (-0.03, 1.34) | ⨁⨁◯◯ LOW |
|  | The rate of nausea and vomiting | OS | Integrated Traditional Chinese and Western Medicine | WM | 2 (172) | RR=0.915, 95%CI (0.267, 3.138) | ⨁⨁◯◯ LOW |
|  | The rate of diarrhea | OS | Integrated Traditional Chinese and Western Medicine | WM | 2 (225) | RR=5.598, 95%CI (0.267, 166.774) | ⨁⨁◯◯ LOW |
|  | The rate of liver damage | OS | Integrated Traditional Chinese and Western Medicine | WM | 2 (202) | RR=0.281, 95%CI (0.046, 1.706) | ⨁⨁◯◯ LOW |
| Sun, et al., 2020 | Clinical effective rate | RCT | (Toujie Quwen Recipe, Shufeng Jiedu capsule) + WM | WM | 2 (273) | RR=1.21, 95%CI (1.08, 1.36) | ⨁⨁⨁◯ MODERATE^*^ |
|  | Adverse reactions | RCT | (Qingfei Touxie Fuzheng Recipe, Jinhua Qinggan granules, Toujie Quwen Recipe, Pneumonia No.1 Prescription, Shufeng Jiedu capsule, Reyanning Mixture) + WM | WM | 7 (681) | RR=1.17, 95%CI (0.39, 3.52) | ⨁⨁◯◯ LOW^*$^ |
|  | Viral nucleic acid negative conversion rate | RCT | (Pneumonia No.1 Prescription, Shufeng Jiedu capsule, Reyanning Mixture) + WM | WM | 3 (185) | RR=1.49, 95%CI (1.13, 1.97) | ⨁⨁⨁◯ MODERATE^*^ |
|  | Remission rate of pulmonary inflammation  (Chest CT) | RCT | (Qingfei Touxie Fuzheng Recipe, Pneumonia No.1 Prescription, Shufeng Jiedu capsule, Reyanning Mixture) + WM | WM | 4 (415) | RR=1.27, 95%CI (1.12, 1.44) | ⨁⨁⨁◯ MODERATE^*^ |
|  | White blood cell count | RCT | (Toujie Quwen Recipe, Pneumonia No.1 Prescription, Shufeng Jiedu capsule) + WM | WM | 4 (415) | MD=0.92, 95%CI (0.07, 1.76) | ⨁⨁◯◯ LOW^*$^ |
|  | Lymphocyte count | RCT | (Toujie Quwen Recipe, Pneumonia No.1 Prescription, Reyanning Mixture) + WM | WM | 3 (339) | MD=0.33, 95%CI (0.08, 0.57) | ⨁⨁◯◯ LOW^*$^ |
|  | Lymphocyte ratio | RCT | (Toujie Quwen Recipe, Shufeng Jiedu capsule) + WM | WM | 3 (188) | MD=2.90, 95%CI (2.09, 3.71) | ⨁⨁⨁◯ MODERATE^*^ |
|  | The amount of C-reactive protein | RCT | (Qingfei Touxie Fuzheng Recipe, Touxie Quwen Recipe, Pneumonia No.1 Prescription, Reyanning Mixture) + WM | WM | 2 (273) | MD=-12.66, 95%CI (-24.40, -0.92) | ⨁⨁◯◯ LOW^*$^ |
|  | IL-6 levels | RCT | (Qingfei Touxie Fuzheng Recipe, Pneumonia No.1 Prescription) + WM | WM | 4 (288) | MD=-8.17, 95%CI (-22.40, 6.06) | ⨁⨁◯◯ LOW^*$^ |
| Pang, et al., 2020 | Number of patients turned to severe and critical type | RCT | (Jinhua Qinggan granules, Toujie Quwen granules, Lianhua Qingwen granules, Maxing Xuanfei Jiedu Decoction, Lianhua Qingke granules) + WM | WM | 8 (988) | RR=0.47, 95%CI (0.32, 0.69) | ⨁⨁⨁◯ MODERATE^*^ |
|  | All-cause death | RCT | (Lianhua Qingwen granules, Chinese Herbal Medcine) + WM | WM | 2 (337) | RR=0.50, 95%CI (0.08, 3.00) | ⨁⨁⨁◯ MODERATE^*^ |
|  | Length of hospital stay | RCT | Qingfeipaidu decoction + WM | WM | 1 (12) | MD=-7.95, 95%CI (-14.66, -1.24) | ⨁⨁◯◯ LOW^*&^ |
|  | Rate of 2019-nCov RT-PCR turned to negativity | RCT | Lianhua Qingwen granules + WM | WM | 1 (284) | RR=1.08, 95%CI (0.94, 1.24) | ⨁⨁⨁◯ MODERATE^*^ |
|  | Defervescence time | RCT | (Maxing Xuanfei Jiedu Decoction, Shufeng Jiedu capsule) + WM | WM | 2 (250) | MD=-1.20, 95%CI (-2.03, -0.38) | ⨁⨁◯◯ LOW^*$^ |
|  | Defervescence rate | RCT | (Qingfei Touxie Fuzheng Recipe, Jinhua Qinggan granules, Lianhua Qingke granules) + WM | WM | 3 (232) | RR=1.18, 95%CI (0.88, 1.60) | ⨁⨁◯◯ LOW^*$^ |
|  | Cough resolution time | RCT | (Maxing Xuanfei Jiedu Decoction, Shufeng Jiedu capsule) + WM | WM | 2 (250) | MD=-1.57, 95%CI (-4.17, 1.03) | ⨁⨁◯◯ LOW^*$^ |
|  | Cough resolution rate | RCT | (Qingfei Touxie Fuzheng Recipe, Jinhua Qinggan granules, Lianhua Qingke granules) + WM | WM | 3 (264) | RR=1.37, 95%CI (1.15, 1.64) | ⨁⨁⨁◯ MODERATE^*^ |
|  | Fatigue resolution time | RCT | Shufeng Jiedu capsule + WM | WM | 1 (200) | MD=-0.33, 95%CI (-0.78, 0.12) | ⨁⨁⨁◯ MODERATE^*^ |
|  | Fatigue resolution rate | RCT | (Jinhua Qinggan granules, Lianhua Qingke granules) + WM | WM | 2 (147) | RR=1.37, 95%CI (1.02, 1.83) | ⨁⨁⨁◯ MODERATE^*^ |
|  | Shortless breath resolution rate | RCT | Qingfei Touxie Fuzheng Recipe + WM | WM | 1 (35) | RR=2.20, 95%CI (1.11, 4.39) | ⨁⨁◯◯ LOW^*&^ |
|  | Diarrhea resolution rate | RCT | (Qingfei Touxie Fuzheng Recipe, Jinhua Qinggan granules) + WM | WM | 2 (30) | RR=0.32, 95%CI (0.01, 15.49) | ⨁⨁◯◯ LOW^*$^ |
|  | Body pain resolution rate | RCT | Jinhua Qinggan granules + WM | WM | 1 (30) | RR=1.17, 95%CI (0.73, 1.87) | ⨁⨁◯◯ LOW^*&^ |
|  | Adverse events | RCT | (Qingfei Touxie Fuzheng Recipe, Jinhua Qinggan granules, Toujie Quwen granules, Lianhua Qingwen granules, Qingfeipaidu decoction, Shufeng Jiedu capsule) + WM | WM | 8 (1152) | RR=0.03, 95%CI (-0.02, 0.08) | ⨁⨁◯◯ LOW^*&^ |
| Luo, et al., 2020 | Overall clinical effectiveness | RCT + OS | (Lianhua Qingwen granules, Shufeng Jiedu capsule, Toujie Quwen granules, Reyanning Mixture, Xuebijing Injection, Qingfeipaidu decoction, Jinhua Qinggan granules) + WM | WM | 10 (812) | OR=2.67, 95%CI (1.83, 3.89) | ⨁⨁⨁◯ MODERATE^*^ |
|  | The rate of CT improvement | RCT + OS | (Lianhua Qingwen granules, Reyanning Mixture, Jiaweidayuan decoction, Xuebijing Injection, Qingfei Touxie Fuzheng Recipe, Shufeng Jiedu capsule, Toujie Quwen granules, Pneumonia No.1 Prescription, Qingfeipaidu decoction) + WM | WM | 13 (955) | OR=2.43, 95%CI (1.80, 3.29) | ⨁⨁⨁◯ MODERATE^*^ |
|  | Percentage of cases turning to severe/critical | RCT + OS | (Toujie Quwen granules, Pneumonia No.1 Prescription, Qingfeipaidu decoction, Lianhua Qingwen granules, Qingfei Touxie Fuzheng Recipe, Reyanning Mixture) + WM | WM | 9 (840) | OR=0.40, 95%CI (0.24, 0.67) | ⨁⨁⨁◯ MODERATE^*^ |
|  | RT-PCR negativity rate | OS | (Shufeng Jiedu capsule, Reyanning Mixture, Pneumonia No.1 Prescription, Jiaweidayuan decoction, Xuebijing Injection, Jinhua Qinggan granules) + WM | WM | 5 (312) | OR=2.55, 95%CI (1.06, 6.17) | ⨁⨁⨁◯ MODERATE^#^ |
|  | The disappearance rate of fever | RCT + OS | (Lianhua Qingwen granules, Jinhua Qinggan granules, Qingfei Touxie Fuzheng Recipe) + WM | WM | 5 (468) | OR=3.17, 95%CI (1.95, 5.15) | ⨁⨁⨁◯ MODERATE^*^ |
|  | The disappearance rate of cough | RCT + OS | (Lianhua Qingwen granules, Jinhua Qinggan granules, Qingfei Touxie Fuzheng Recipe) + WM | WM | 5 (468) | OR=2.95, 95%CI (1.88, 4.63) | ⨁⨁⨁◯ MODERATE^*^ |
|  | The disappearance rate of fatigue | RCT + OS | (Lianhua Qingwen granules, Jinhua Qinggan granules) + WM | WM | 5 (468) | OR=2.61, 95%CI (1.56, 4.34) | ⨁⨁⨁◯ MODERATE^*^ |
|  | Length of hospital stay | OS | (Reyanning Mixture, Qingfeipaidu decoction, Xuebijing Injection, Jiaweidayuan decoction) + WM | WM | 5 (272) | WMD=-0.46, 95%CI (-3.87, 2.95) | ⨁⨁◯◯ LOW |
|  | Adverse effects | RCT + OS | (Shufeng Jiedu capsule, Pneumonia No.1 Prescription, Jinhua Qinggan granules, Reyanning Mixture, Qingfeipaidu decoction, Jiaweidayuan decoction, Xuebijing Injection, Qingfei Touxie Fuzheng Recipe, Toujie Quwen granules, Jinhua Qinggan granules) + WM | WM | 8 (714) | OR=1.21, 95%CI (0.48, 3.07) | ⨁⨁⨁◯ MODERATE^*^ |
| Jin, et al., 2020 | Clinical efficacy | RCT | Qingfei Touxie Fuzheng Recipe + WM | WM | 2 (100) | OR=2.25, 95%CI (1.01, 5.01) | ⨁⨁⨁◯ MODERATE^*^ |
|  | Clinical efficacy | RCT | Lianhua Qingwen granules + WM | WM | 3 (397) | OR=1.38, 95%CI (0.91, 2.08) | ⨁⨁⨁◯ MODERATE^*^ |
|  | Clinical efficacy | RCT | Lianhua Qingke granules + WM | WM | 2 (82) | OR=12.06, 95%CI (1.37, 106.04) | ⨁⨁◯◯ LOW^*&^ |
|  | Clinical efficacy | RCT | Xuebijing Injection + WM | WM | 2 (44) | OR=9.80, 95%CI (1.09, 88.23) | ⨁⨁◯◯ LOW^*&^ |
| Hu, et al., 2020 | The improvement rate of fever | RCT | Lianhua Qingwen + WM | WM | 4 (322) | OR=4.05, 95%CI (1.78, 6.59) | ⨁⨁⨁◯ MODERATE^*^ |
|  | The improvement rate of cough | RCT | Lianhua Qingwen + WM | WM | 4 (322) | OR=3.43, 95%CI (1.87, 6.29) | ⨁⨁⨁◯ MODERATE^*^ |
|  | The improvement rate of fatigue | RCT | Lianhua Qingwen + WM | WM | 4 (322) | OR=2.82, 95%CI (1.44, 5.53) | ⨁⨁⨁◯ MODERATE^*^ |
|  | The improvement rate of muscle pain | RCT | Lianhua Qingwen + WM | WM | 4 (322) | OR=5.01, 95%CI (1.44, 17.40) | ⨁⨁⨁◯ MODERATE^*^ |
|  | The rate of improvement sore throat | RCT | Lianhua Qingwen + WM | WM | 4 (322) | OR=0.31, 95%CI (0.03, 2.99) | ⨁⨁⨁◯ MODERATE^*^ |
|  | The improvement rate of nausea | RCT | Lianhua Qingwen + WM | WM | 4 (322) | OR=1.98, 95%CI (0.49, 7.95) | ⨁⨁⨁◯ MODERATE^*^ |
|  | The improvement rate of diarrhoea | RCT | Lianhua Qingwen + WM | WM | 4 (322) | OR=1.28, 95%CI (0.18, 9.30) | ⨁⨁⨁◯ MODERATE^*^ |
|  | The improvement rate of loss of appetite | RCT | Lianhua Qingwen + WM | WM | 4 (322) | OR=6.05, 95%CI (0.69, 53.14) | ⨁⨁◯◯ LOW^*$^ |
|  | The improvement rate of sputum | RCT | Lianhua Qingwen + WM | WM | 3 (438) | OR=4.30, 95%CI (1.01, 18.22) | ⨁⨁◯◯ LOW^*$^ |
|  | The rate improvement of shortness of breath | RCT | Lianhua Qingwen + WM | WM | 3 (438) | OR=10.62, 95%CI (3.71, 30.40) | ⨁⨁⨁◯ MODERATE^*^ |
|  | The improvement rate of breathlessness | RCT | Lianhua Qingwen + WM | WM | 3 (438) | OR=4.81, 95%CI (0.97, 23.86) | ⨁⨁⨁◯ MODERATE^*^ |
|  | The improvement rate of chest tightness | RCT | Lianhua Qingwen + WM | WM | 3 (438) | OR=3.02, 95%CI (1.23, 7.42) | ⨁⨁⨁◯ MODERATE^*^ |
|  | The improvement rate of pulmonary imaging | RCT | Lianhua Qingwen + WM | WM | 5 (697) | OR=1.77, 95%CI (1.29, 2.42) | ⨁⨁⨁◯ MODERATE^*^ |
|  | Curative effect | RCT | Lianhua Qingwen + WM | WM | 5 (697) | OR=2.49, 95%CI (1.76, 3.53) | ⨁⨁⨁◯ MODERATE^*^ |
|  | Healing period | RCT | Lianhua Qingwen + WM | WM | 2 (119) | WMD=-2.06, 95%CI (-3.36, -0.75) | ⨁⨁⨁◯ MODERATE^*^ |
|  | The rate conversion of severe cases | RCT | Lianhua Qingwen + WM | WM | 2 (346) | OR=0.46, 95%CI (0.27, 0.77) | ⨁⨁⨁◯ MODERATE^*^ |
| Fan, et al., 2020 | The level of C-reactive protein | RCT | (Qingfei Touxie Fuzheng Recipe, Toujie Quwen granules, Maxinshigan-dayuanyin decoction, Shenfu decoction, Jiaweidayuanyin decoction) + WM | WM | 5 (325) | MD=-11.82, 95%CI (-17.95, -5.69) | ⨁⨁◯◯ LOW^*$^ |
|  | The improvement rate of lung CT | RCT | (Qingfei Touxie Fuzheng Recipe, Toujie Quwen granules, Jiaweidayuanyin decoction) + WM | WM | 4 (489) | RR=1.34, 95%CI (1.19, 1.51) | ⨁⨁⨁◯ MODERATE^*^ |
| Ouyang, et al., 2021 | Overall effective rate | RCT + OS | (Toujie Quwen granules, Shufeng Jiedu capsule, Lianhua Qingwen capsule, Lianhua Qingwen granules) + WM | WM | 4 (435) | RR=1.26, 95%CI (1.14, 1.40) | ⨁⨁⨁◯ MODERATE^*^ |
|  | Fever reduction time | RCT + OS | (Reyanning Mixture, Shufeng Jiedu capsule, Lianhua Qingwen granules) + WM | WM | 5 (463) | WMD=-1.21, 95%CI (-1.71, -0.71） | ⨁⨁⨁◯ MODERATE^*^ |
|  |  | RCT | (Reyanning Mixture, Shufeng Jiedu capsule) + WM | WM | 2 (249) | WMD=-0.85, 95%CI (-1.22, -0.47) | ⨁⨁⨁◯ MODERATE^*^ |
|  |  | OS | (Lianhua Qingwen granules, Shufeng Jiedu capsule) + WM | WM | 3 (214) | WMD=-1.46, 95%CI (-2.11, -0.80) | ⨁⨁◯◯ LOW |
|  | Absorption rate of pneumonia | RCT + OS | (Reyanning Mixture, Toujie Quwen granules, Shufeng Jiedu capsule, Lianhua Qingwen granules) + WM | WM | 6 (504) | RR=1.15, 95%CI (0.93, 1.43） | ⨁⨁◯◯ LOW^*$^ |
|  | Viral nucleic acid conversion | RCT + OS | (Reyanning Mixture, Lianhua Qingwen granules, Shufeng Jiedu capsule) + WM | WM | 3 (221) | RR=1.47,95%CI (1.05，2.05) | ⨁⨁⨁◯ MODERATE^*^ |
|  | Disappearance rate of fever | RCT + OS | (Reyanning Mixture, Jinhua Qinggan granules, Lianhua Qingwen granules, Shufeng Jiedu capsule) + WM | WM | 6 (487) | RR=1.25,95%CI (0.88, 1.80) | ⨁⨁◯◯ LOW^*$^ |
|  | Disappearance rate of fatigue | RCT + OS | (Jinhua Qinggan granules, Lianhua Qingwen granules, Shufeng Jiedu capsule) + WM | WM | 5 (438) | RR=1.36,95%CI (0.71, 2.62) | ⨁⨁◯◯ LOW^*$^ |
|  | Disappearance rate of cough | RCT + OS | (Jinhua Qinggan granules, Lianhua Qingwen granules, Shufeng Jiedu capsule) + WM | WM | 5 (438) | RR=1.87, 95%CI (0.58, 6.08) | ⨁⨁◯◯ LOW^*$^ |
|  | White blood cell count | RCT | (Toujie Quwen granules, Shufeng Jiedu capsule) + WM | WM | 2 (273) | RR=0.74, 95%CI (0.26, 1.22) | ⨁⨁◯◯ LOW^*$^ |
|  | Lymphocyte count | RCT | (Reyanning Mixture, Toujie Quwen granules) + WM | WM | 2 (122) | RR=0.21, 95%CI (0.15, 0.27） | ⨁⨁⨁◯ MODERATE^*^ |
|  | Percentage of lymphocytes | RCT | (Toujie Quwen granules, Shufeng Jiedu capsule) + WM | WM | 2 (273) | MD=2.69, 95%CI (1.92, 3.47) | ⨁⨁⨁◯ MODERATE^*^ |
|  | Adverse effects | RCT + OS | (Jinhua Qinggan granules, Shufeng Jiedu capsule) + WM | WM | 3 (393) | RR=1.94, 95%CI (0.14, 26.21) | ⨁⨁◯◯ LOW^*$^ |
|  | The rate of aggravated pneumonia | RCT + OS | (Jinhua Qinggan granules, Toujie Quwen granules, Lianhua Qingwen granules) + WM | WM | 4 (399) | RR=0.40, 95%CI (0.23, 0.70) | ⨁⨁⨁◯ MODERATE^*^ |
| Liu, et al., 2021 | The rate of ranging from mild to critical | RCT + OS | (Lianhua Qingwen granules, Jinhua Qinggan granules) + WM | WM | 3 (326) | OR=0.35, 95%CI (0.18, 0.69) | ⨁⨁⨁◯ MODERATE^*^ |
|  | Overall effective rate | OS | (Lianhua Qingwen granules, Jinhua Qinggan granules, Shufeng Jiedu capsule) + WM | WM | 3 (346) | OR=2.50, 95%CI (1.46, 4.29) | ⨁⨁⨁◯ MODERATE^#^ |
|  | CT improvement rate | OS | (Lianhua Qingwen granules, Jinhua Qinggan granules, Shufeng Jiedu capsule) + WM | WM | 3 (346) | OR=2.27, 95%CI (1.37, 3.77) | ⨁⨁⨁◯ MODERATE^#^ |
|  | Fever reduction time | OS | (Lianhua Qingwen granules, Shufeng Jiedu capsule) + WM | WM | 4 (414) | SMD=-0.81, 95%CI (-1.25, -0.38) | ⨁⨁◯◯ LOW |
|  | Disappearance rate of fever | RCT + OS | (Lianhua Qingwen granules, Jinhua Qinggan granules) + WM | WM | 4 (343) | OR=3.05, 95%CI (1.85, 5.01) | ⨁⨁⨁◯ MODERATE^*^ |
|  | Disappearance rate of cough | RCT + OS | (Lianhua Qingwen granules, Jinhua Qinggan granules) + WM | WM | 4 (322) | OR=2.99, 95%CI (1.84, 4.85) | ⨁⨁⨁◯ MODERATE^*^ |
|  | Disappearance rate of fatigue | RCT + OS | (Lianhua Qingwen granules, Jinhua Qinggan granules) + WM | WM | 4 (283) | OR=2.60, 95%CI (1.56, 4.33) | ⨁⨁⨁◯ MODERATE^*^ |
|  | Disappearance rate of sputum | RCT + OS | (Lianhua Qingwen granules, Jinhua Qinggan granules) + WM | WM | 4 (315) | OR=1.94, 95%CI (1.19, 3.18) | ⨁⨁◯◯ LOW^*$^ |
| Gao, et al., 2021 | Total effectiveness of treatment | RCT | Toujie Quwen granules + WM | WM | 2 (138) | RR=1.313, 95% CI (1.105, 1.560) | ⨁⨁⨁◯ MODERATE^*^ |
|  | The control rate of fever | RCT + OS | (Lianhua Qingwen granules, Pneumonia No.1 Prescription, Jinhua Qinggan granules, Qingfei Touxie Fuzheng Recipe) + WM | WM | 6 (536) | RR=1.296, 95% CI (1.161, 1.447) | ⨁⨁⨁◯ MODERATE^*^ |
|  | The score of fever | RCT + OS | (Reyanning Mixture, Touxie Quwen granules) + WM | WM | 3 (177) | SMD=0.762, 95%CI (-0.571, 2.095) | ⨁⨁⨁◯ MODERATE^*^ |
|  |  | RCT | Toujie Quwen granules + WM | WM | 2 (128) | SMD=1.457, 95%CI (1.080, 1.833) | ⨁⨁⨁◯ MODERATE^*^ |
|  |  | OS | Reyanning Mixture + WM | WM | 1 (49) | SMD=-0.638, 95% CI (-1.214, -0.063) | ⨁⨁◯◯ LOW |
|  | Fever reduction time | OS | (Lianhua Qingwen granules, Shufeng Jiedu capsule) + WM | WM | 5 (333) | WMD=-1.577, 95% CI (-1.980, -1.173) | ⨁⨁◯◯ LOW |
|  | The improvement rate of fatigue | RCT + OS | (Lianhua Qingwen granules, Jinhua Qinggan granules) + WM | WM | 4 (368) | RR=1.553, 95% CI (1.213, 1.990) | ⨁⨁⨁◯ MODERATE^*^ |
|  | The score of fatigue | RCT + OS | (Reyanning Mixture, Touxie Quwen granules) + WM | WM | 3 (187) | SMD=1.489, 95% CI (0.679, 2.300) | ⨁⨁⨁◯ MODERATE^*^ |
|  |  | RCT | (Reyanning Mixture, Touxie Quwen granules) + WM | WM | 2 (122) | SMD=1.432, 95%CI (0.137, 2.727) | ⨁⨁◯◯ LOW^*$^ |
|  |  | OS | Touxie Quwen granules + WM | WM | 1 (65) | SMD=1.623, 95%CI (0.973, 2.273) | ⨁⨁◯◯ LOW |
|  | Fatigue reduction time | OS | (Lianhua Qingwen granules, Shufeng Jiedu capsule) + WM | WM | 2 (172) | WMD=-1.742, 95%CI (-2.006, -1.478) | ⨁⨁◯◯ LOW |
|  | The improvement rate of cough | RCT + OS | (Lianhua Qingwen capsule, Jinhua Qinggan granules, Qingfei Touxie Fuzheng Recipe) + WM | WM | 5 (468) | RR=1.653, 95%CI (1.341, 2.036) | ⨁⨁⨁◯ MODERATE^*^ |
|  | The score of coughs | RCT + OS | (Reyanning Mixture, Toujie Quwen granules) + WM | WM | 3 (187) | SMD=1.951, 95% CI (1.127, 2.774) | ⨁⨁◯◯ LOW^*$^ |
|  |  | RCT | Toujie Quwen granules + WM | WM | 2 (138) | SMD=2.307, 95%CI (1.465, 3.150) | ⨁⨁◯◯ LOW^*$^ |
|  |  | OS | Reyanning Mixture + WM | WM | 1 (49) | SMD=1.226, 95%CI (0.613, 1.839) | ⨁⨁◯◯ LOW |
|  | Cough reduction time | OS | (Lianhua Qingwen granules, Shufeng Jiedu capsule) + WM | WM | 2 (172) | WMD= -1.711, 95%CI (-2.303, -1.119) | ⨁⨁◯◯ LOW |
|  | CT improvement rate | RCT + OS | (Lianhua Qingwen granules, Pneumonia No.1 Prescription, Jinhua Qinggan granules, Reyanning Mixture, Toujie Quwen granules) + WM | WM | 7 (526) | RR=1.278, 95%CI (1.040, 1.571) | ⨁⨁⨁◯ MODERATE^*^ |
|  |  | RCT | (Jinhua Qinggan granules, Toujie Quwen granules) + WM | WM | 2 (188) | RR=1.385, 95%CI (1.083, 1.771) | ⨁⨁⨁◯ MODERATE^*^ |
|  |  | OS | (Lianhua Qingwen granules, Pneumonia No.1 Prescription, Reyanning Mixture) + WM | WM | 5 (338) | RR=1.252, 95%CI (0.955, 1.642) | ⨁⨁◯◯ LOW |
|  | Viral nucleic acid negative conversion rate | OS | (Pneumonia No.1 Prescription, Shufeng Jiedu capsule) + WM | WM | 2 (138） | RR=1.426, 95%CI (0.939, 2.164) | ⨁⨁◯◯ LOW |
|  | The rate of ranging from mild to critical | RCT + OS | (Lianhua Qingwen granules, Pneumonia No.1 Prescription, Jinhua Qinggan granules, Qingfei Touxie Fuzheng Recipe, Reyanning Mixture, Shufeng Jiedu capsule, Toujie Quwen granules) + WM | WM | 10 (803) | RR=0.438, 95%CI (0.258, 0.673) | ⨁⨁⨁◯ MODERATE^*^ |
| Zhang, et al., 2020 | Clinical efficacy | RCT | (Lianhua Qingwen granules, Lianhua Qingwen capsule) + WM | WM | 3 (457) | RR＝2.59, 95%CI (1.68,3.98) | ⨁⨁⨁◯ MODERATE^*^ |
|  | Disappearance rate of fever | RCT | Lianhua Qingwen granules + WM | WM | 3 (220) | RR=3.43, 95%CI (1.78, 6.59) | ⨁⨁⨁◯ MODERATE^*^ |
|  | Disappearance rate of cough | RCT | Lianhua Qingwen granules + WM | WM | 3 (189) | RR=3.46, 95%CI (1.85, 6.49) | ⨁⨁⨁◯ MODERATE^*^ |
|  | Disappearance rate of fatigue | RCT | Lianhua Qingwen granules + WM | WM | 3 (131) | RR=2.65, 95%CI (1.07, 6.58) | ⨁⨁⨁◯ MODERATE^*^ |
|  | Disappearance rate of shortness of breath | RCT | Lianhua Qingwen granules + WM | WM | 3 (457) | RR=13.68, 95%CI (2.80, 66.79) | ⨁⨁⨁◯ MODERATE^*^ |
|  | Disappearing time of fever | RCT | Lianhua Qingwen granules + WM | WM | 2 (103) | MD=-1.07, 95%CI (-1.77, -0.37) | ⨁⨁⨁◯ MODERATE^*^ |
|  | Adverse effects | RCT | Lianhua Qingwen granules + WM | WM | 2 (203) | RR=0.33, 95%CI (0.13, 0.81) | ⨁⨁⨁◯ MODERATE^*^ |
| Zhang, et al., 2020 | Disappearance rate of fever | RCT | Lianhua Qingwen + WM | WM | 4 (229) | RR=1.33, 95%CI (1.13, 1.56) | ⨁⨁⨁◯ MODERATE^*^ |
|  | Disappearance rate of cough | RCT | Lianhua Qingwen + WM | WM | 4 (256) | RR=1.77, 95%CI (1.36, 2.28) | ⨁⨁⨁◯ MODERATE^*^ |
|  | Disappearance rate of fatigue | RCT | Lianhua Qingwen + WM | WM | 4 (184) | RR=1.46, 95%CI (1.16, 1.86) | ⨁⨁⨁◯ MODERATE^*^ |
|  | The rate of ranging from mild to critical | RCT | Lianhua Qingwen + WM | WM | 4 (555) | RR=0.51, 95%CI (0.34, 0.77) | ⨁⨁⨁◯ MODERATE^*^ |
| Yang, et al., 2020 | Disappearance rate of fever | RCT | Lianhua Qingwen + WM | WM | 3 (251) | RR=1.76, 95% CI (1.05, 2.96) | ⨁⨁◯◯ LOW^*$^ |
|  | Disappearance rate of cough | RCT | Lianhua Qingwen + WM | WM | 3 (251) | RR=1.96, 95% CI (1.43, 2.68) | ⨁⨁⨁◯ MODERATE^*^ |
|  | Disappearance rate of fatigue | RCT | Lianhua Qingwen + WM | WM | 3 (251) | RR=1.77, 95% CI (1.36, 2.30) | ⨁⨁⨁◯ MODERATE^*^ |
|  | Disappearance rate of chest tightness | RCT | Lianhua Qingwen + WM | WM | 3 (251) | RR=2.19, 95% CI (0.89, 5.40) | ⨁⨁◯◯ LOW^*$^ |
|  | Disappearance rate of difficulty breathing | RCT | Lianhua Qingwen + WM | WM | 3 (251) | RR=4.58, 95% CI (2.39, 8.79) | ⨁⨁⨁◯ MODERATE^*^ |
|  | Disappearance rate of loss of appetite | RCT | Lianhua Qingwen + WM | WM | 3 (251) | RR=1.36, 95% CI (1.00, 1.84) | ⨁⨁⨁◯ MODERATE^*^ |
| Wu, et al., 2020 | The rate of ranging from mild to critical | OS | (Herbal decoction, Chinese patent medicine, Chinese herb injection) + WM | WM | 2 (163) | RR=0.18, 95%CI (0.04, 0.79) | ⨁⨁◯◯ LOW |
|  | Clinical cure rate | OS | (Herbal decoction, Chinese patent medicine, Chinese herb injection, Shufeng Jiedu granules) + WM | WM | 3 (189) | RR=7.82, 95%CI (2.04, 30.06) | ⨁⨁⨁◯ MODERATE^#^ |
|  | Case fatality rate | OS | (Herbal decoction, Chinese patent medicine, Chinese herb injection) + WM | WM | 2 (163) | RR=0.18, 95%CI (0.01, 4.23) | ⨁⨁◯◯ LOW |
|  | Disappearing time of fever | RCT + OS | (Herbal decoction, Chinese patent medicine, Chinese herb injection, Shufeng Jiedu granules, Lianhua Qingwen capsule) + WM | WM | 4 (231) | SMD=-0.81, 95%CI (-1.53, -0.09) | ⨁⨁◯◯ LOW^*$^ |
|  | The rate of cough remission | RCT + OS | (Qingfei Touxie Fuzheng Recipe, Lianhua Qingwen capsule, Shufeng Jiedu granules) + WM | WM | 3 (212) | RR=5.14, 95%CI (1.49, 17.75) | ⨁⨁⨁◯ MODERATE^*^ |
|  | The rate of diarrhea remission | RCT + OS | (Qingfei Touxie Fuzheng Recipe, Lianhua Qingwen capsule, Shufeng Jiedu granules) + WM | WM | 3 (212) | RR=1.19, 95%CI (0.17, 8.64) | ⨁⨁⨁◯ MODERATE^*^ |
|  | The absorption rate of pneumonia | RCT + OS | (Qingfei Touxie Fuzheng Recipe, Shufeng Jiedu granules) +WM | WM | 3 (219) | RR=1.02, 95%CI (0.85, 1.22) | ⨁⨁⨁◯ MODERATE^*^ |
| Wang, et al., 2020 | CT recovery rate | RCT | (Lianhua Qingwen granules, Shiduyufei decoction, Huoxiang zhengqi oral liquid, Lianhua Qingwen capsule, Shufeng Jiedu capsule, Shuangshuanglian oral liquid, Yupingfeng granules) + WM | WM | 5 (573) | RR=1.14, 95%CI (1.02, 1.28) | ⨁⨁◯◯ LOW^*$^ |
|  | The rate of ranging from mild to critical | RCT | (Lianhua Qingwen granules, Lianhua Qingwen capsule, Shiduyufei decoction, Huoxiang zhengqi oral liquid + WM | WM | 4 (271) | RR=0.48, 95%CI (0.31, 0.72) | ⨁⨁⨁◯ MODERATE^*^ |
|  | Fever duration | RCT | (Lianhua Qingwen granules, Lianhua Qingwen capsule, Shiduyufei decoction, Huoxiang zhengqi oral liquid + WM | WM | 3 (186) | SMD=-0.87, 95%CI (-1.22, -0.52) | ⨁⨁⨁◯ MODERATE^*^ |
|  | Hospital stays | RCT | (Lianhua Qingwen capsule, Lianhua Qingwen granules, Shiduyufei decoction, Huoxiang zhengqi oral liquid, Shufeng Jiedu capsule, Shuangshuanglian oral liquid, Yupingfeng granules) + WM | WM | 4 (234) | SMD=-0.61, 95%CI (-0.91, -0.30) | ⨁⨁⨁◯ MODERATE^*^ |
| Qi, et al., 2020 | Effective rate | RCT + OS | Lianhua Qingwen granules + WM | WM | 3 (260) | RD=0.83, 95%CI (0.72, 0.95) | ⨁⨁◯◯ LOW^*$^ |
|  | Disappearance rate of fever | RCT + OS | Lianhua Qingwen granules + WM | WM | 4 (281) | RD=0.88, 95%CI (0.78, 0.98) | ⨁⨁◯◯ LOW^*$^ |
|  | Disappearance rate of cough | RCT + OS | Lianhua Qingwen granules + WM | WM | 4 (281) | RD=0.67, 95%CI (0.57, 0.78) | ⨁⨁⨁◯ MODERATE^*^ |
|  | Disappearance rate of fatigue | RCT + OS | Lianhua Qingwen granules + WM | WM | 4 (281) | RD=0.69, 95%CI (0.54, 0.84) | ⨁⨁◯◯ LOW^*$^ |
|  | Disappearance rate of loss of appetite | RCT + OS | Lianhua Qingwen granules + WM | WM | 3 (226) | RD=0.34, 95%CI (0.20, 0.47) | ⨁⨁⨁◯ MODERATE^*^ |
|  | Disappearance rate of sputum | RCT | Lianhua Qingwen granules + WM | WM | 2 (72) | RD=0.59, 95%CI (0.42, 0.75) | ⨁⨁⨁◯ MODERATE^*^ |
|  | Disappearance rate of chest tightness | RCT + OS | Lianhua Qingwen granules + WM | WM | 4 (281) | RD=0.72, 95%CI (0.58, 0.85) | ⨁⨁⨁◯ MODERATE^*^ |
| Cai, et al., 2020 | Overall effective rate | RCT + OS | Lianhua Qingwen granules + WM | WM | 2 (175) | RR=1.27, 95%CI (1.08, 1.49) | ⨁⨁⨁◯ MODERATE^*^ |
|  | Cure rate | RCT + OS | (Toujie Quwen granules, Shufeng Jiedu capsule) + WM | WM | 3 (195) | RR=1.59, 95%CI (1.12, 2.25) | ⨁⨁⨁◯ MODERATE^*^ |
|  | Disappearance rate of fever | RCT + OS | (Jinhua Qinggan granules, Lianhua Qingwen granules) + WM | WM | 3 (224) | RR=1.45, 95%CI (1.19, 1.76) | ⨁⨁⨁◯ MODERATE^*^ |
|  | Disappearance rate of cough | RCT + OS | (Jinhua Qinggan granules, Lianhua Qingwen granules) + WM | WM | 3 (199) | RR=1.83, 95%CI (1.31, 2.55) | ⨁⨁⨁◯ MODERATE^*^ |
|  | Disappearance rate of fatigue | RCT + OS | (Jinhua Qinggan granules, Lianhua Qingwen granules) + WM | WM | 3 (175) | RR=1.54, 95%CI (1.14, 2.09) | ⨁⨁⨁◯ MODERATE^*^ |
|  | Disappearing time of fever | OS | (Lianhua Qingwen granules, Shufeng Jiedu capsule) + WM | WM | 4 (248) | MD=-1.52, 95%CI (-1.91, -1.14) | ⨁⨁◯◯ LOW |
|  | Disappearing time of cough | OS | (Lianhua Qingwen granules, Shufeng Jiedu capsule) + WM | WM | 2 (146) | MD=-1.63, 95%CI (-2.16, -1.10) | ⨁⨁◯◯ LOW |
|  | Disappearing time of fatigue | OS | (Lianhua Qingwen granules, Shufeng Jiedu capsule) + WM | WM | 2 (136) | MD=-1.71, 95%CI (-1.97, -1.46) | ⨁⨁◯◯ LOW |
|  | CT recovery rate | OS | (Lianhua Qingwen granules) + WM | WM | 2 (181) | RR=1.26, 95%CI (0.96, 1.65) | ⨁⨁◯◯ LOW |
|  | The rate of ranging from mild to critical | RCT + OS | (Toujie Quwen granules, Lianhua Qingwen granules) + WM | WM | 3 (193) | RR=0.32, 95%CI (0.15, 0.69) | ⨁⨁⨁◯ MODERATE^*^ |
|  | Adverse effects | RCT + OS | Toujie Quwen granules + WM | WM | 2 (135) | RR=3.07, 95%CI (0.03, 341.61) | ⨁⨁◯◯ LOW^*$^ |

^*^The risk of bias (random, distributive hiding or blind) was a factor of downgrading; ^$^The heterogeneity test *P* value was very small, and the I square was larger were downgrading factors; ^&^The imprecision with small sample, and wide confidence interval were downgrading factors; ^#^Larger effect was an upgrading factor. RCT, Randomized controlled trial; OS, Observational study.
